# Global sensitivity analysis of integrated assessment models with multivariate outputs

**DOI:** 10.1111/risa.70002

**Published:** 2025-02-22

**Authors:** Leonardo Chiani, Emanuele Borgonovo, Elmar Plischke, Massimo Tavoni

**Affiliations:** ^1^ Department of Management, Economics and Industrial Engineering Politecnico di Milano Milan Italy; ^2^ CMCC Foundation ‐ Euro‐Mediterranean Center on Climate Change Milan Italy; ^3^ RFF‐CMCC European Institute on Economics and the Environment Milan Italy; ^4^ Department of Decision Sciences and Bocconi Institute for Data Science and Analytics Bocconi University Milan Italy; ^5^ Institute of Resource Ecology Helmholtz‐Zentrum Dresden‐Rossendorf Dresden Germany

**Keywords:** climate change, global sensitivity analysis, integrated assessment models, uncertainty quantification

## Abstract

Risk assessments of complex systems are often supported by quantitative models. The sophistication of these models and the presence of various uncertainties call for systematic robustness and sensitivity analyses. The multivariate nature of their response challenges the use of traditional approaches. We propose a structured methodology to perform uncertainty quantification and global sensitivity analysis for risk assessment models with multivariate outputs. At the core of the approach are novel sensitivity measures based on the theory of optimal transport. We apply the approach to the uncertainty quantification and global sensitivity analysis of emissions pathways estimated via an eminent open‐source climate–economy model (RICE50+). The model has many correlated inputs and multivariate outputs. We use up‐to‐date input distributions and long‐term projections of key demographic and socioeconomic drivers. The sensitivity of the model is explored under alternative policy architectures: a cost‐benefit analysis with and without international cooperation and a cost‐effective analysis consistent with the Paris Agreement objective of keeping temperature increase below 2°C. In the cost‐benefit scenarios, the key drivers of uncertainty are the emission intensity of the economy and the emission reduction costs. In the Paris Agreement scenario, the main driver is the sensitivity of the climate system, followed by the projected carbon intensity. We present insights at the multivariate model output level and discuss how the importance of inputs changes across regions and over time.

## 1 INTRODUCTION

In risk assessment applications, analysts and engineers frequently rely on quantitative models. This reliance is particularly significant in climate risk assessment: projecting the evolution of climate‐related quantities becomes imperative to design effective mitigation policies as environmental risks intensify. This is a complex task and, as summarized in the latest IPCC assessment report (Intergovernmental Panel On Climate Change, 2023), researchers employ a variety of methods to obtain reliable projections, with numerical models for policy evaluation playing a central role. These models have grown in importance and complexity, making their validation and auditing more relevant than ever (Saltelli et al., 2020; Saltelli & Di Fiore, 2023). Coupled climate–economy models typically have many inputs, often uncertain or poorly known. This uncertainty propagates to the output, making their projections uncertain as well. Thus, proper uncertainty quantification and sensitivity analysis become paramount (Saltelli et al., 2020) to help resolve ongoing controversies over which emission projections should be used as reference (Hausfather & Peters, 2020) and to build trust in the numerical tools used to inform climate policies. A central challenge in this task is that complex models often produce multivariate outputs, either due to the spatial or temporal nature of the analysis or because multiple quantities are relevant to policy‐makers in such a multifaceted decision‐making process.

Few works have built a bridge between sensitivity analysis and climate change mitigation research. Anderson et al. (2014) and Butler et al. (2014) highlighted the importance of comprehensive global sensitivity analysis (GSA) in integrated assessment modeling. Bosetti et al. (2015) exposed the relevance of future technology costs inside some detailed process models, and Marangoni et al. (2017) tested the assumptions in shared socioeconomic pathways narratives across multiple models. Recently, Lamontagne et al. (2019) used sensitivity analysis to examine robust mitigation pathways. Other works explored more advanced approaches for simple climate–economy models, such as Bayesian calibration of the input distributions (Srikrishnan et al., 2022), meta‐modeling (Miftakhova, 2021), and mixture probability measures for the inputs (Borgonovo et al., 2022).

However, these studies focused on univariate quantities of interest, and a systematic approach to multivariate responses is missing. For instance, suppose the output of interest is ${\mathrm{CO}}_{2}$ emissions. If the approach can deal only with one output at a time, the analyst would have to consider cumulative emissions over time or the emissions at a specific point in time. This is unsatisfactory as decision‐makers are interested in the entire time profile of the emissions. Moreover, complex coupled climate–economy models have regional or spatial inputs that can be highly correlated. The presence of correlations represents a significant challenge for the use of methods that require input independence.

To address these issues, we propose a methodology that allows risk analysts to perform systematic uncertainty and sensitivity analysis of complex models, considering the multivariate nature of their output, the correlated nature of their inputs and conduct mixed scenario and global sensitivity analyses. We also strive to use methods that are computationally efficient. To address input correlation and computational complexity, we rely on recently introduced GSA methods based on the theory of optimal transport (Borgonovo et al., 2024). They allow for a systematic examination of the model's response to variations in correlated inputs across multiple dimensions, providing a comprehensive understanding of input importance. Moreover, these indices can be estimated directly from the available input–output dataset, without requiring specific sampling schemes. This approach minimizes the number of model evaluations, which is especially advantageous when working with computationally expensive models like coupled climate–economy ones. To illustrate the possibilities offered by the proposed methodology, we apply it to the open‐source coupled climate–economy model, RICE50+ (Gazzotti, 2022).

We organize the article as follows. Section 2 presents a literature review regarding uncertainty quantification of integrated assessment models and multivariate GSA. Section 3 explains the GSA methods and algorithms exploited in the analysis. Section 4 presents the workflow and the goals of the analysis. Section 5 contains the application of the methodology to RICE50+, with the results and main points of interest. Section 6 summarizes the findings and main contributions.

## 2 RELATED LITERATURE

Uncertainty quantification and GSA have a long tradition in risk analysis (Helton & Davis, 2002; Saltelli, 2002). Uncertainty analysis concerns the propagation of input variability to assess the output variability (Helton & Davis, 2002; Iman & Helton, 1988). We usually rely on Monte Carlo simulations: the analyst generates a set of input realizations from the given distributions and correspondingly evaluates the model response(s). We gain insights into the distribution of the outputs, their mean values, quantiles, and other quantities of interest to the decision‐maker. In the climate–economy literature, we recall the uncertainty analyses performed on the Dynamic Integrated model of Climate and the Economy (DICE) model (Nordhaus, 2014) and more recently in a multimodel comparison (Gillingham et al., 2018). Rennert, Errickson, et al. (2022) perform a comprehensive exploration of the uncertainty in the social cost of ${\mathrm{CO}}_{2}$ using the greenhouse gas impact value estimator model. Srikrishnan et al. (2022) calibrate a simple model on historical data and produce probabilistic projections of important socioeconomic variables.

Global sensitivity analysis goes a step further yielding insights into the key drivers of model variability (factor prioritization) (Saltelli, 2002), on the marginal response of the output on a given input (direction of change) and the presence of interactions (interaction quantification) (Borgonovo & Plischke, 2016). We refer to Saltelli (2002) and Borgonovo (2006) for early reviews and to Razavi et al. (2021) for a more recent overview. This last work shows notable progress and a flourishing of techniques in recent years, of which the risk assessment, particularly the climate modeling literature, has benefited only partly. Existing works studied either simple models (Anderson et al., 2014; Butler et al., 2014; Lamontagne et al., 2019; Miftakhova, 2021) or only a small subset of inputs (Bosetti et al., 2015; Marangoni et al., 2017). The climate modeling community mainly focused on model granularity: models provide a detailed representation of the behaviors exhibited by climate, economy, and energy production. However, these efforts risk backfiring if modelers do not perform sensitivity analysis, leaving out the quantification of uncertainty. Indeed, an increased model complexity may lead to higher uncertainty, requiring careful investigation and calibration before drawing policy‐relevant conclusions from the outputs (Puy et al., 2022). The climate modeling community is becoming more aware of this pressing issue, as shown in a recent perspective paper by Guivarch et al. (2022). Moreover, existing literature (apart from Lamontagne et al., 2019 that use sensitivity maps) focuses on scalar targets. This focus may severely limit the insights of the analysis. First, outputs such as emission paths are inherently time‐dependent. Moreover, models often have regional or spatial output, adding another dimension. It can be challenging to find a precise measure that effectively summarizes these results.

At the same time, research addressing multivariate responses in GSA is ongoing. We find two main approaches. Marrel et al. (2016) describe sensitivity analysis maps. The intuition is to perform a separate sensitivity analysis of each univariate part of the output. This approach is straightforward and benefits from the extensive advances in the GSA of individual outputs. However, in the case of a multiplicity of outputs, results might be difficult to summarize to a decision maker. Alternatively, one can rely on approaches developed to consider the multivariate output as a single quantity. We recall the variance‐based approach of Lamboni et al. (2011) and Gamboa et al. (2014), which represent an extension of Sobol' indices to the multivariate output case. We also recall new indexes based on machine learning techniques, such as reproducing kernel Hilbert spaces (Barr & Rabitz, 2022, 2023; Da Veiga, 2015, 2021; Deb et al., 2020) and optimal transport (Borgonovo et al., 2024; Wiesel, 2022). We focus on the second set of methods for their properties and applicability.

## 3 GLOBAL SENSITIVITY ANALYSIS

### 3.1 The common rationale

We consider an analyst in charge of evaluating one or more quantities of interest $Y=\{Y_{1},\cdots ,Y_{p}\}$ in a given risk analysis problem. The analyst expresses these quantities of interest as a function of a set of inputs $X=(X_{1},\cdots ,X_{k})$. To accomplish this task, the analyst builds or has available a quantitative model that links the inputs to the outputs. We write

(1) Y=f(X),

where f is the model input–output mapping. Let X be the support of the inputs, that is $X\subseteq X_{1}\times X_{2}\times \cdots \times X_{k}$, with $X_{i}\in X_{i}$, for i=1,2,⋯,k. Let Y be the image of X under f, so that f(X)∈Y for all values of X. Then, if the model inputs are known and fixed at $X=\tilde{x}$, the quantities of interest are also known, and they equal at $\tilde{Y}=f\left(\tilde{x}\right)$. However, in most situations, we cannot fix the inputs at a given value because we do not know the value, or they may have no fixed value. We then regard them as random variables with some known distribution.

In this framework, the outputs Y also become random variables and their distribution is determined by propagating uncertainty via the input–output mapping in Equation (1). More technically, the inputs and outputs are random vectors on the same probability space (Ω,B,P). We denote their distributions by $P_{X}\in P\left(X\right)$ and $P_{Y}\in P\left(Y\right)$, where P(X) and P(Y) are the sets of probability distributions on X and Y, respectively. These distributions represent the current degree of belief of the decision‐maker about the inputs and outputs. Note that no specific assumption is posed on the input distributions: they can be dependent or independent, continuous or discrete (without changing the theory or the mathematical results in this section). The following example helps us illustrate the notation.

Example 1 Consider a problem in which an analyst is modeling two quantities of interest as a function of three inputs using a linear model. We have the output $Y=\{Y_{1},Y_{2}\}$ and the input $X=(X_{1},X_{2},X_{3})$, with $Y=g\left(X_{1},X_{2},X_{3}\right)=AX^{T}$, where we specifically assign:

(2) $$\left\{\begin{array}{c} Y_{1}=4X_{1}-2X_{2}+X_{3}\\ Y_{2}=2X_{1}+5X_{2}-X_{3}. \end{array}\right.$$

Suppose that uncertainty in the inputs is reflected by a multivariate normal distribution, with mean $m_{X}=\left(1,1,1\right)$, and variance–covariance matrix $\Sigma_{X}=\left(\begin{array}{ccc} 1 & 0.5 & 0.5\\ 0.5 & 1 & 0.5\\ 0.5 & 0.5 & 1 \end{array}\right)$. Then, this uncertainty propagates to Y, which becomes a normal random vector with mean $m_{Y}=\left(3,6\right)$ and variance–covariance matrix $\Sigma_{Y}=\left(\begin{array}{cc} 15 & 7.5\\ 7.5 & 33 \end{array}\right)$.

Consider assessing which input impacts uncertainty in Y the most. This analysis is usually performed by assuming that the decision‐maker is informed that the ith input $X_{i}$ has some fixed value $x_{i}$. This new information modifies the output distribution $P_{Y}$ into the conditional distribution $P_{Y|X_{i}=x_{i}}$. Indeed, we can interpret $P_{Y}$ as the model output distribution reflecting the current degree of belief of the decision‐maker about Y and $P_{Y|X_{i}=x_{i}}$ as reflecting the degree of belief once $X_{i}$ is fixed at $x_{i}$. We then define a function γ, called inner statistic, that quantifies the change in the degree of belief about Y given the new information about $X_{i}$. The inner statistic is then defined as (Borgonovo et al., 2016)

(3) $$\gamma \left(X_{i}\right)=\zeta \left(P_{Y},P_{Y|X_{i}}\right),$$

where ζ(·,·) is a separation measurement between probability distributions, with the property that ζ(P,P)=0, that is, ζ(·,·) is equal to zero when the two involved distributions are identical. Because $X_{i}$ is random, $\gamma (X_{i})$ is random. Taking the expected value with respect to $X_{i}$, we obtain the index:

(4) $$\xi \left(Y,X_{i}\right)=E\left[\zeta \left(P_{Y},P_{Y|X_{i}}\right)\right]=E\left[\gamma \left(X_{i}\right)\right],$$

called the global sensitivity measure of $X_{i}$ with respect to Y. To illustrate, let us consider scalar input–output mapping. In this case, it means Y=g(X). Let us set

(5) $$\gamma \left(X_{i}\right)=\text{Var}\left(Y\right)-\text{Var}\left(Y|X_{i}\right).$$

Taking the expectation with respect to $X_{i}$, we obtain the variance‐based importance measure introduced by Iman and Hora (1990), which equals $\xi^{IH}\left(Y,X_{i}\right)=\text{Var}\left(Y\right)-E\left[\text{Var}\left(Y|X_{i}\right)\right]$. Indeed, the normalized expected value of γ with respect to $X_{i}$ is

(6) $$\xi^{V}\left(Y,X_{i}\right)=\eta_{i}^{2}=\frac{\text{Var}(E\left[Y|X_{i}\right])}{\text{Var}(Y)},$$

which corresponds to Pearson's correlation ratio (Pearson, 1905) and the first‐order Sobol' indices (Sobol', 2001).

Alternatively, suppose that Y is a continuous random variable, with marginal and conditional probability density functions given, respectively, by $f_{Y}\left(y\right)$ and $f_{Y|X_{i}}\left(y\right)$. If we set the inner statistic equal to the Kullback–Leibler divergence between $P_{Y}$ and $P_{Y|X_{i}}$

(7) $$\gamma^{KL}\left(X_{i}\right)=\mathrm{KL}\left(P_{Y|X_{i}}|P_{Y}\right)=\int \log\left(\frac{f_{Y|X_{i}}\left(y\right)}{f_{Y}\left(y\right)}\right)f_{Y|X_{i}}\left(y\right)dy,$$

we find the mutual information between Y and $X_{i}$ as a global importance measure (Soofi, 1994). We have

(8) $$\begin{array}{c} E\left[\gamma^{KL}\left(X_{i}\right)\right]=\int_{X_{i}}f_{X_{i}}\left(x_{i}\right)\int \log\left(\frac{f_{Y,X_{i}}\left(y,x_{i}\right)}{f_{Y}\left(y\right)}\right)f_{Y|X_{i}}\left(y;x_{i}\right)dydx_{i}\\ =\int_{X_{i}}\int \log\left(\frac{f_{Y,X_{i}}\left(y,x_{i}\right)}{f_{Y}f_{X_{i}}\left(y,x_{i}\right)}\right)f_{Y,X_{i}}\left(y,x_{i}\right)dydx_{i}=\text{MI}\left(Y,X_{i}\right). \end{array}$$

There are several other examples, comprising, among others, the well‐known δ importance measure or the recently introduced kernel‐based importance measure of Barr & Rabitz (2022). Relevant to the choice in this paper is the recent literature that has renewed interest in the desirable properties for measures of statistical association. In particular, the works of Chatterjee (2021) and Wiesel (2022) outline the following two. The first property is zero‐independence, corresponding to Rényi's postulate D (Rényi, 1959). This axiom requires that a measure of statistical association is equal to zero if and only if Y and $X_{i}$ are statistically independent. In this way, the analyst is sure that a null value of the sensitivity measure signifies that learning $X_{i}$ does not affect the distribution of Y. A second property is max‐functionality, stating that a measure of statistical association reaches its maximum value when learning $X_{i}$ resolves uncertainty in Y. Not all the global sensitivity measures discussed above possess these two properties. For instance, variance‐based sensitivity measures in Equation (6) do not satisfy zero independence but comply with max‐functionality. We illustrate this intuition in the following example.

Example 2 Consider the following input–output mapping: $Y=X_{1}X_{2}+X_{3}$, with $X_{1}$ standard normal, and $X_{2}$ and $X_{3}$ normally distributed with mean value and standard deviation equal to 1. For this model, we have $\xi^{V}\left(Y,X_{1}\right)=0.32$, $\xi^{V}\left(Y,X_{2}\right)=0$, and $\xi^{V}\left(Y,X_{3}\right)=0.33$. Note that the variance‐based importance of the second input is null, although the output actually depends on $X_{2}$. The reason is that fixing $X_{2}$ does not change the expected value of Y.

Variance‐based indices possess max‐functionality. Suppose we fix all model inputs at $\tilde{X}=\tilde{x}$. Then, we have that Y becomes a unique value, (we have $\tilde{Y}=g\left(\tilde{x}\right)$) with zero variance. Inserting into Equation (6), we find $E[\text{Var}\left(Y|X_{1},X_{2},X_{3}\right)]=0$, so that $\xi^{V}\left(Y,X_{1},X_{2},X_{3}\right)=1$. Conversely, the mutual information between Y and $X_{i}$ satisfies zero‐independence, but not max‐functionality. That is, a null value of $\text{MI}(Y,X_{i})$ reassures us that Y is independent of $X_{i}$. However, $\text{MI}(Y,X_{i})$ reaches its maximum value (which equals infinity) even if learning $X_{i}$ does not resolve uncertainty in Y completely. For instance, one records a mutual information equal to infinity if learning $X_{i}$ makes the support of Y change from continuous to discrete. To remedy this issue, we select a separation measurement based on the theory of optimal transport that allows us to obtain both zero‐independence and max‐functionality as properties of the resulting global sensitivity measure. We discuss our choice in the next section.

### 3.2 Global sensitivity analysis using optimal transport

Optimal transport is a well‐known research subject across mathematics and statistics (see Figalli & Glaudo, 2021. for an overview). Relevant to this work is that the formulation of the problem leads to a distance between distributions known as the Wasserstein metric. This distance can be expressed as follows (see Borgonovo et al., 2024, for details):

(9)

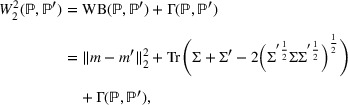

where m, $m^{\prime }$ and Σ, $\Sigma^{\prime }$ are the means and covariance matrices of P and $P^{\prime }$, respectively, and Tr is the trace operator, summing all diagonal entries of a square matrix. The sum of the first two terms on the right‐hand side is defined as Wasserstein–Bures semimetric and is defined by the term WB(·,·). The first term in this metric is the optimal cost for matching the mean values of P and $P^{\prime }$, that is, their first‐order moments. The second term is the optimal cost for matching their covariance matrices, that is, their second‐order moments. The third term Γ(·,·) is the residual cost for matching any higher order moment for which P and $P^{\prime }$ differ. We call it a residual term and observe that, generally, it has no analytical representation. Therefore, it is not generally possible to write the Wasserstein distance between two generic distributions in closed form. However, there is a special case in which the residual term is null. As proven in Gelbrich (1990), if the two involved distributions are elliptical with the same characteristic generator, then the residual term is null, and the Wasserstein distance is completely determined by the sum of the first two terms in Equation (9). The family of elliptical distributions contains the normal, Student‐*t*, and logistic distributions among others. We refer to the works of Cambanis et al. (1981) and Landsman & Valdez (2003) for a treatment of their statistical properties. In a GSA context, setting $P=P_{Y}$ and $P^{\prime }=P_{Y|X_{i}=x_{i}}$, we can define the inner statistics based on the 2‐Wasserstein distance as

(10) $$\gamma^{W_{2}^{2}}\left(X_{i}\right)=W_{2}^{2}\left(P_{Y},P_{Y|X_{i}}\right).$$

We then call

(11) $$\xi^{W_{2}^{2}}\left(Y,X_{i}\right):=\int_{X}W_{2}^{2}\left(P_{Y},P_{Y|X_{i}=x_{i}}\right)dP_{X_{i}}$$

the optimal‐transport‐based global sensitivity measure of $X_{i}$ with respect to Y. It is possible to show that $\xi^{W_{2}^{2}}\left(Y,X_{i}\right)\geq 0$ and that has an upper bound given by $M^{W_{2}^{2}}\left[Y\right]:=2V\left[Y\right]$, where V[Y] is the sum of the diagonal elements of the variance–covariance matrix of the output(s) Y. Because $M^{W_{2}^{2}}\left[Y\right]>0$, it is then natural to introduce the normalized optimal transport sensitivity index (OT index, henceforth)

(12) $$\iota^{W_{2}^{2}}\left(Y,X_{i}\right)=\frac{\xi^{W_{2}^{2}}\left(Y,X_{i}\right)}{2V[Y]}.$$

It is possible to see that $\iota^{W_{2}^{2}}\left(Y,X_{i}\right)=0$ if and only if $X_{i}$ and Y are independent. Also, it is $\iota^{W_{2}^{2}}\left(Y,X_{i}\right)=1$ if and only if Y is a deterministic function of $X_{i}$. Thus, $\iota^{W_{2}^{2}}\left(Y,X_{i}\right)$ possesses the zero independence property as well as max‐functionality. Using Equation (9), we obtain the following decomposition of $\iota^{W_{2}^{2}}\left(Y,X_{i}\right)$:

(13) $$\begin{array}{ccc} \iota^{W_{2}^{2}}\left(Y,X_{i}\right) & = & \iota^{V}\left(Y,X_{i}\right)+\iota^{\Sigma }\left(Y,X_{i}\right)+E\left[\Gamma \left(P_{Y},P_{Y|X_{i}}\right)\right]\\ & = & \iota^{\text{WB}}\left(Y,X_{i}\right)+E\left[\Gamma \left(P_{Y},P_{Y|X_{i}}\right)\right], \end{array}$$

where

(14) $$\iota^{\text{WB}}\left(Y,X_{i}\right)=\iota^{V}\left(Y,X_{i}\right)+\iota^{\Sigma }\left(Y,X_{i}\right).$$

In Equations (13) and (14), $\iota^{V}\left(Y,X_{i}\right)$ is the variance‐based contribution, $\iota^{\Sigma }\left(Y,X_{i}\right)$ is the co‐variance‐based contribution (both of them form the Wasserstein–Bures contribution) and $E[\Gamma \left(P_{Y},P_{Y|X_{i}}\right)]$ is the residual contribution of higher order moments. The interpretation of Equation (13) is as follows: the first summand quantifies the expected impact of learning $X_{i}$ on the mean value of Y, the second the expected impact on the variance–covariance matrix, and the third term accounts for the impact of higher order moments. Let us analyze these terms in greater detail, starting with $\iota^{V}\left(Y,X_{i}\right)$. This index can be written as

(15) $$\iota^{V}\left(Y,X_{i}\right)=\frac{1}{2V[Y]}E\left[\text{Tr}\left(\Sigma_{Y}-\Sigma_{Y|X_{i}}\right)\right]=\frac{1}{2}\iota^{LG}\left(Y,X_{i}\right),$$

where $\iota^{LG}\left(Y,X_{i}\right)$ is the generalized variance‐based importance measure introduced in Lamboni et al. (2011) and Gamboa et al. (2014). Thus, $\iota^{V}\left(Y,X_{i}\right)$ links the optimal‐transport approach to the previously introduced generalized variance‐based sensitivity measures.

The covariance matrix contribution $\iota^{\Sigma }\left(Y,X_{i}\right)$ equals

(16) $$\begin{array}{ccc} \iota^{\Sigma }\left(Y,X_{i}\right) & = & \frac{1}{2V[Y]}E\left[\text{Tr}\left(\Sigma_{Y}+\Sigma_{Y|X_{i}}-2{\left(\Sigma_{Y}^{1/2}\Sigma_{Y|X_{i}}\Sigma_{Y}^{1/2}\right)}^{1/2}\right)\right]\\ & = & 1-\iota^{V}\left(Y,X_{i}\right)-\frac{1}{V[Y]}E\left[\text{Tr}{\left(\Sigma_{Y}^{1/2}\Sigma_{Y|X_{i}}\Sigma_{Y}^{1/2}\right)}^{1/2}\right]. \end{array}$$

Equation (16) shows that new information about an input allows us to directly quantify its impact on the covariance matrix, independent of its influence on other statistical moments. We illustrate the meaning of the indices by continuing our running example.

Example 1 (continued) Consider the model $Y=f(X_{1},X_{2},X_{3})$ described in Example 1. Since both X and Y are normally distributed, $\iota \left(Y,X_{i}\right)=\iota^{WB}\left(Y,X_{i}\right)$. The risk analysts are interested in the most important inputs in her problem, giving full consideration to input uncertainty. The analysts may consider the outputs $Y_{1}$ and $Y_{2}$ separately, forming a so‐called sensitivity map, or simultaneously. In the first case, they can calculate the variance‐based importance measures $\eta^{2}\left(Y^{j},X^{i}\right)$, j∈{1,2} separately for each output. We report the results in Table 1.The results show that the most important input for $Y_{1}$ is $X_{1}$, while this output is almost not affected by $X_{2}$, which is the key driver of uncertainty in $Y_{2}$. In the second case, the risk analysts can consider simultaneously the two outputs and evaluate the input importance using $\iota_{2}^{W_{2}^{2}}\left(Y,X_{i}\right)$. The indices can be calculated in closed form using Equation (14). Table 2 reports the results illustrating the variance and covariance matrix contributions.In this case, the overall picture is clearer. $X_{2}$ is the most important input, followed closely by $X_{1}$. Moreover, we can study how the inputs change the output mean and covariance separately. The fourth column in Table 2 shows that the variance‐based parts amount to 60% and 63% of the OT index of $X_{1}$ and $X_{2}$, respectively, and at about 90% of the importance of $\iota (Y,X_{3})$. Therefore, both the terms play a relevant role in defining the inputs' importance.

**TABLE 1:** Variance‐based importance measures for Example 1.

	$\eta^{2}\left(\cdot ,X_{1}\right)$	$\eta^{2}\left(\cdot ,X_{2}\right)$	$\eta^{2}\left(\cdot ,X_{3}\right)$	$\sum_{i=1}^{3}\eta^{2}\left(\cdot ,X_{i}\right)$
$Y_{1}$	0.82	0.02	0.27	1.11
$Y_{2}$	0.48	0.92	0.19	1.29

**TABLE 2:** $\iota (Y,X_{i})$, and associated decompositions into variance‐based and covariance‐based parts for the model in Equation (2).

	$\iota^{WB}\left(Y,X_{i}\right)$	$\iota^{V}$	$\iota^{\Sigma }$	$\iota^{V}/\iota^{WB}$
$X_{1}$	0.492	0.294	0.198	60%
$X_{2}$	0.507	0.318	0.189	63%
$X_{3}$	0.117	0.107	0.01	91%

### 3.3 Estimation

Estimation in a computationally feasible time is crucial for the applicability of any global sensitivity method. The brute force estimation of global sensitivity measures in Equation (4) requires a double‐batch of Monte Carlo simulations. The first batch is needed to compute the conditional distribution $P_{Y|X_{i}}$, and the second is an iteration over all possible values of $X_{i}$. These operations need to be repeated for each input $X_{i}$. The procedure results in a computational cost quadratic in the sample size and linear in the problem dimension, say $C^{\text{Brute Force}}=N^{2}\times k$, where N is the sample size and k is the number of inputs.

The cost is then rapidly increasing in N. Because of the dependence of k, it is exposed to the curse of dimensionality. With k equal to 10 and N to 100, we reach 100,000 runs, which becomes 1,000,000 with k = 100. However, several approaches have been developed in the literature to reduce such computational burden. We recall the pick‐and‐freeze method (Gamboa et al., 2016), which makes the cost linear in both k and N. However, this cost can be further decreased by using a given‐data approach that implies a single batch of Monte Carlo simulations and whose computational cost is N model runs (Röhlig et al., 2009; Strong et al., 2015). A given‐data approach can be traced back to the intuition of Pearson regarding the estimation of first‐order variance‐based sensitivity indices. Let $X_{i}$ be the support of input $X_{i}$ and consider a partition of $X_{i}$ formed by H subsets, $X_{i}^{h}$ h∈{1,⋯,H}. Then, we can define an estimator for any global sensitivity measure in the common rationale as

(17) $$\hat{\xi }\left(Y,X_{i};N,H\right)=\frac{1}{H}\sum_{h=1}^{H}\zeta \left(P_{Y}^{N},P_{Y|X_{i}\in X_{i}^{h}}^{N}\right).$$

In Equation (17), the symbol $P^{N}$ denotes an empirical distribution or a statistic calculated from the N realizations. The condition $X_{i}=x_{i}$ in Equation (12) is replaced by the more general condition $X_{i}\in X_{i}^{h}$. The sum is taken over all partitions, and it results in a discretization of the expectation in Equation (4) when $X_{i}$ is a continuous random variable. An estimate in Equation (17) also requires a suitable algorithm for the calculation of $\zeta (P_{Y}^{N},P_{Y|X_{i}\in X_{i}^{h}}^{N})$.

When the global sensitivity measure is based on optimal transport, we need to calculate the quantity

(18) $$\hat{\xi }\left(Y,X_{i};N,H\right)=\frac{1}{H}\sum_{h=1}^{H}W_{2}^{2}\left(P_{Y}^{N},P_{Y|X_{i}\in X_{i}^{h}}^{N}\right),$$

which implies the solution of an optimal transport problem between $P_{Y}^{N}$ and $P_{Y|X_{i}\in X_{i}^{h}}^{N}$ for each partition. An inner separation based on optimal transport has some advantages from the estimation viewpoint. The calculation of Wasserstein distances does not require an accurate estimation of probability densities or cumulative distribution functions. The theory of optimal transport states that $W_{2}^{2}\left(P_{Y}^{N},P_{Y|X_{i}\in X_{i}^{h}}^{N}\right)$ can be found by solving a corresponding linear program based solely on the data available from the sample. If Y is a scalar (p=1), the solution of the optimal transport problem is straightforward, as the only required operation is a reordering of the data, which makes estimation very convenient also for large problem sizes. If Y is a multidimensional vector, then one needs to solve a data‐driven linear program. For this task, several efficient algorithms are made available by a rich body of work in machine learning and mathematics (Peyré & Cuturi, 2020; Chizat et al., 2020). In our experiments, we rely on the entropic approximation of the optimal transport problem, introduced in an influential work by Cuturi (2013). The entropic approximation allows the use of the fixed‐point iteration algorithm introduced by Sinkhorn (1967), using only efficient matrix‐scalar operations.

An estimate of the Wasserstein–Bures index in Equation (13) is written as

(19) $${\hat{\iota }}^{WB}\left(Y,X_{i}\right)={\hat{\iota }}^{V}\left(Y,X_{i}\right)+{\hat{\iota }}^{\Sigma }\left(Y,X_{i}\right),$$

In a given‐data context, we have the given‐data estimates of $\iota^{V}\left(Y,X_{i}\right)$ and $\iota^{\Sigma }\left(Y,X_{i}\right)$ equal, respectively, to

(20) $${\hat{\iota }}^{V}\left(Y,X_{i}\right)=\frac{1}{2\hat{V}\left[Y\right]}\sum_{h=1}^{H}\frac{N_{h}}{N}\sum_{t=1}^{p}{\left({\hat{m}}_{Y_{t}}-{\hat{m}}_{Y_{t}|X_{i}\in X_{i}^{h}}\right)}^{2}$$

and for the covariance‐based part is

(21) $$\begin{array}{ccc} & & {\hat{\iota }}^{\Sigma }\left(Y,X_{i}\right)\\ & & =\frac{1}{2\hat{V}\left[Y\right]}\sum_{h=1}^{H}\frac{N_{h}}{N}\text{Tr}\left({\hat{\Sigma }}_{Y}+{\hat{\Sigma }}_{Y|X_{i}\in X_{i}^{h}}-2{\left({\hat{\Sigma }}_{Y}^{1/2}{\hat{\Sigma }}_{Y|X_{i}\in X_{i}^{h}}{\hat{\Sigma }}_{Y}^{1/2}\right)}^{1/2}\right). \end{array}$$

In the previous expressions, ${\hat{m}}_{Y_{t}}$ and ${\hat{m}}_{Y_{t}|X_{i}\in X_{i}^{h}}$ are empirical means and ${\hat{\Sigma }}_{Y}$ and ${\hat{\Sigma }}_{Y|X_{i}\in X_{i}^{h}}$ are empirical covariance matrices. $N_{h}$ is the number of observations in the hth partition. These expressions involve only algebraic operations, and they are computationally fast.

Overall, the following algorithmic procedure is followed in our work. We first generate a sample of size N of input–output realizations $\left\{\left(x^{\left(1\right)},y^{\left(1\right)}\right),\left(x^{\left(2\right)},y^{\left(2\right)}\right),\cdots ,\left(x^{\left(N\right)},y^{\left(N\right)}\right)\right\}$. Then, for each input $X_{i}$, we form the scatterplot between $X_{i}$ and Y, sorting the sample according to the values of $X_{i}$. We then partition the horizontal axis of the scatterplot into a set of H equally populated bins. For each bin, we calculate the squared Wasserstein distance. Taking the average over the H partitions results in the estimate $\hat{\xi }\left(Y,X_{i};N,H\right)$.

## 4 THE METHODOLOGY

**FIGURE 1:**
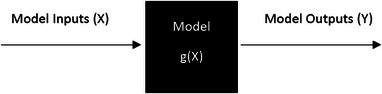
A black box representation of a computer simulation.

**FIGURE 2:**
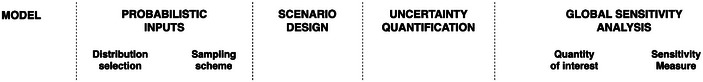
The steps of our sensitivity analysis methodology.

In sensitivity analysis, we take a black box approach, in which the model is not necessarily known analytically but rather encoded in a computer program. The model is then a machine that processes the inputs to calculate a set of quantities of interest (Figure 1). We consider steps to help analysts interrogate the model and to increase their awareness of its behavior and transparency in their inferences. Following Saltelli: “[…] a sensitivity analysis should not focus on the model output as such, but rather on the answer the model is supposed to provide, on the thesis that it is supposed to prove or disprove.” (Saltelli, 2002, p. 582) Sensitivity analysis thus serves also as a systematic way to assess the robustness and the adequacy of the model response. Figure 2 offers a high‐level representation of the steps of our approach, inspired by Pianosi et al. (2016) and Borgonovo (2023). Given the model, the first step is to identify the inputs to be subjected to the sensitivity analysis. We may need to distinguish two groups: a set of uncertain inputs to which it may be appropriate to assign probability distributions (probabilistic inputs, henceforth) and a set of inputs that can be fixed at predetermined scenarios (parameters, henceforth). The choice depends critically on the context and research question. A key criterion is how the choice helps build a narrative (an internally consistent story). For example, in nuclear waste management, the soil type may be treated as a parameter if the analyst wishes to isolate the effect of distinct terrains.

The next task is to assign distributions to the probabilistic inputs. This delicate task has received much attention in the risk analysis literature (Apostolakis, 1990, 2004). Concisely, we can identify three main approaches: (a) the adoption of distributions discussed in the literature, (b) the use of expert judgment to assign distributions to inputs not previously covered (Oppenheimer et al., 2016), and (c) the reliance on principles that acknowledge the lack of information, such as the Jeffreys noninformative priors or the Laplace's principle (Gilboa & Marinacci, 2016). We note that the probability distributions may be conditional on the value of the parameters in each scenario.

We can then perform an uncertainty quantification in each scenario. In this step, the analyst obtains insights on the variability of the model output determining the relevant statistical properties from knowledge of its distribution, such as the quantiles, the mean, and the variance. A core element of this step is the sampling scheme. The choice ranges from crude Monte Carlo simulation to designs with better space‐filling properties, such as Latin hypercube sampling (Helton & Davis, 2002), and quasi Monte Carlo schemes using low‐discrepancy sequences (Dick & Pillichshammer, 2010; Dick et al., 2013). While crude Monte Carlo is simple to implement, using space‐filling designs may improve estimation accuracy for a fixed computational budget.

The next step is sensitivity analysis. Here, the selected method may depend on whether the analyst can afford an additional model that runs beyond the ones used for uncertainty quantification. Computational time constraints may suggest a given‐data approach to exploit the already available uncertainty quantification sample (see Subsection 3.3). We consider this choice in our methodology.

A challenge we address is the presence of multivariate quantities of interest (QoIs). The dimensionality can arise from different sources: one source is the dependence of outputs on specific parameters, such as space or time. For example, ${\mathrm{CO}}_{2}$ emissions projections may vary over geographic regions or evolve over time. Another source of multidimensionality reflects the analyst's need to examine multiple aspects of the system's behavior simultaneously. For instance, decision‐makers may wish to assess the interplay between ${\mathrm{CO}}_{2}$ emissions and carbon price in a given year. In both cases, the choice of sensitivity measures that apply to both a univariate and a multivariate output allows us to decide the granularity of the analysis based on the specific needs of decision‐makers. This flexibility is not possible with methods that work only with scalar QoIs. However, it is essential to emphasize that these two levels of analysis are complementary rather than mutually exclusive. Measures for multivariate outputs provide a synthetic representation of the inputs' importance, delivering an overall summary of the system response to input uncertainties. Sensitivity measures for univariate outputs offer a detailed, localized (in time or space) view of the input importance through sensitivity maps over time or regions. This dual perspective ensures that decision‐makers can balance precision and simplicity in their analyses, leveraging the strengths of both approaches to gain deeper insights into the model's behavior.

## 5 APPLICATION: ANALYSIS OF THE EMISSION PATHWAYS IN THE RICE50+ MODEL

**FIGURE 3:**
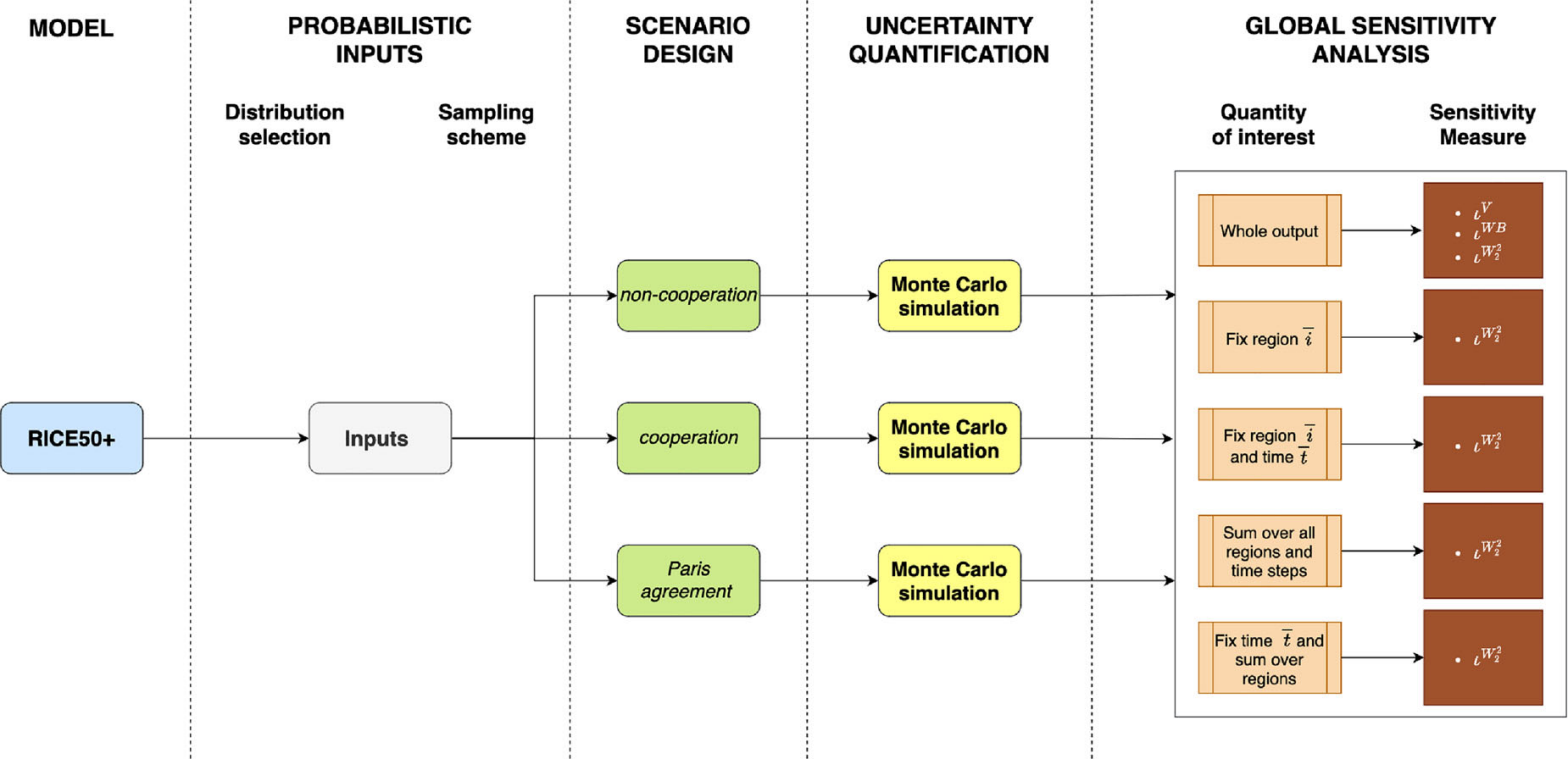
The diagram represents the sensitivity analysis methodology applied to our case study.

We demonstrate the methodology with a real‐world application using the open‐source coupled climate–economy model RICE50+. Figure 3 contextualizes the methodology steps outlined in Figure 2 with the specifics of our case study, detailing the model, scenarios, and quantities of interest. We consider two quantities of interest in the uncertainty quantification: ${\mathrm{CO}}_{2}$ emission pathways and temperature anomaly. ${\mathrm{CO}}_{2}$ emissions are the main focus of our analysis, and temperature anomaly is selected to understand whether the Paris Agreement target is met in any of the scenarios. In GSA, we focus only on ${\mathrm{CO}}_{2}$ emissions pathways for several reasons, rooted both in the nature of integrated assessment models (IAMs) and in precedents set by recent literature on the same topic. First, temperature anomaly, while crucial for public communication, is linearly related to cumulative emissions in the RICE50+ model through the climate system representation. As a result, analyzing emissions provides insights into temperature trajectories as well. Moreover, temperature anomaly is global, removing one important model's dimension. Second, ${\mathrm{CO}}_{2}$ emissions in models like RICE50+ are a direct result of the interaction between economic activity, carbon intensity, and abatement efforts. This makes ${\mathrm{CO}}_{2}$ emissions an effective proxy for studying the impact of abatement decisions across time, as it encompasses the effects of both economic and policy variables while being more understandable by a wide audience not accustomed to IAMs.

In terms of policy relevance, while it is true that actionable variables like the optimal mitigation rate or the social cost of carbon (SCC) are impactful, ${\mathrm{CO}}_{2}$ emissions pathways remain highly informative. Policymakers, international negotiations, and countries usually set net‐zero emissions targets: the emissions trajectories thus provide a concrete connection between mitigation actions and their broader impacts on climate outcomes, including temperature.

Our focus on emissions is also consistent with recent sensitivity analysis studies. Several works have considered emissions (or fossil fuel and industry‐related emissions) as their main QoI. For instance, Bosetti et al. (2015), Marangoni et al. (2017), and Srikrishnan et al. (2022) focus on emissions in their analyses, while Anderson et al. (2014) examine multiple QoIs, with an important focus on ${\mathrm{CO}}_{2}$ emissions and the SCC. Other studies have analyzed different QoIs: Butler et al. (2014) explore the net‐present values of climate damages and abatement costs, and Miftakhova (2021) focuses on the SCC. This diversity of QoIs reflects the complexity of IAMs, which offer multiple important outputs.

After describing the model (Subsection 5.1), we systematically apply the workflow: we define and categorize the inputs (Subsection 5.2), perform uncertainty quantification (Subsection 5.3), and present the results of the sensitivity analysis (Subsection 5.4). In the Appendix, we provide additional results for two different quantities of interest (temperature anomaly in Appendix A and carbon price in Appendix B), and the code implementation details (Appendix C).

### 5.1 The RICE50+ coupled climate–economy model

The model under investigation in this work is RICE50+ (Gazzotti et al., 2021; Gazzotti, 2022). RICE50+ is a generalization of the Regionalized Integrated Model of Climate and the Economy initially developed by Nobel Prize laureate William Nordhaus (Nordhaus & Yang, 1996) as a regionalized extension of the well‐known dynamic integrated model of climate and the economy (DICE) (Nordhaus, 1992). Despite its simplicity, the model continues to inform climate research on a variety of topics (Bastien‐Olvera et al., 2024; Andreoni et al., 2024).

RICE50+ is based on economic and game‐theoretic principles. Its output is the result of a constrained nonlinear optimization of welfare over different regions or countries. In our numerical implementation, we adopt a regional subdivision called “r5,” dividing the world into five parts: Asia (asia), OECD (oecd), Latin America (lam), Middle East and Africa (maf), and Reforming Economies (ref). Regions can cooperate on emission reductions or act independently, optimizing their objectives while interacting with others. Depending on the cooperation option, the model evaluates a noncooperative game equilibrium (Nash equilibrium) or a cooperative game equilibrium. Time is discretized into 58 5‐year steps covering the years from 2015 to 2300.

A complete RICE50+ description can be found in Gazzotti (2022). We outline the main modeling choices and details relevant to our work. We highlight some of the inputs treated as uncertain in our analysis and emphasize their role in model equations.

The objective function in RICE50+ is the welfare function,

(22) $$\begin{array}{ccc} & & W(\mu )\\ & & =\sum_{t=1}^{T}\left\{\frac{1}{1-\eta }{\left[\sum_{i\in N_{c}}w_{pop}\left(t,i\right){\left(\frac{C(t,i,\mu )}{L(t,i)}\right)}^{1-\gamma }\right]}^{\frac{1-\eta }{1-\gamma }}-1\right\}{\left(1+\rho \right)}^{-t+1}, \end{array}$$

where C(t,i,μ) is the total consumption of region i at time t, L(t,i) is the population, $w_{\text{pop}}\left(t,i\right)$ are the weights with values $L\left(t,i\right)/\sum_{j\in N_{c}}L\left(t,j\right)$, η is the intertemporal elasticity of substitution, γ is the inequality aversion, ρ is the discount rate, and $N_{c}$ is the active set of regions to be optimized. In the noncooperative mode, $N_{c}$ is a single region, while in the cooperative one, it corresponds to the entire set of regions, denoted as N. L, η, γ, and ρ are treated as uncertain inputs of our model.

The optimization problem is to maximize the welfare function W over the decision variable μ (the emission control rate), balancing climate change damages, and mitigation costs. For each region i, the value of μ(t,i) is constrained in [0,1] until time period 9 (year 2050). Then, the upper bound linearly increases from 1 to a value M in period $T_{\mu }$. Starting from $T_{\mu }$ and until period 58, μ(t,i) is constrained between 0 and M. This new upper bound M is greater than 1, accounting for the possibility of negative emissions. The growth of the fraction of emissions to abate is also constrained by a parameter Δμ, meaning that μ(t+1,i)≤μ(t,i)+Δμ and μ(t+1,i)≥μ(t,i)−Δμ. The parameters M, $T_{\mu }$, and Δμ are treated as uncertain inputs in our model.

The decision variable μ(t,i) directly affects the emissions from fossil fuels and industry, defined as

(23) $$E_{\text{IND}}\left(t,i\right)=\sigma \left(t,i\right)Y_{\text{GROSS}}\left(t,i\right)\left(1-\mu \left(t,i\right)\right).$$

σ is an exogenous parameter defined as the carbon intensity and couples the gross GDP, $Y_{\text{GROSS}}$, to the industrial emissions $E_{\text{IND}}$. The total emissions ($E_{\text{TOT}}$) in the model are defined as the sum of the emissions from fossil fuels and industry ($E_{\text{IND}}\left(t,i\right)$) in Equation (23) and the emissions from land use ($E_{\text{LU}})\left(t,i\right)$. At the same time, the emissions abatement has a cost depending on the quantity of emissions abated. In the model, the cost of abating 1 $\mathrm{Gt}{\mathrm{CO}}_{2}$ is modeled as

(24) $$\Lambda \left(t,i\right)=\nu \left(t\right)\left[a\left(t,i\right)\frac{\mu {\left(t,i\right)}^{A}}{2}+b\left(t,i\right)\frac{\mu {\left(t,i\right)}^{B}}{5}\right].$$

Since a and b are exogenous and dependent both on time and region, they are high dimensional. To reduce the number of inputs for the sensitivity analysis, we used a sigmoid transition to represent them. This transition describes the shift from initial states $a_{1}\left(i\right)$ and $b_{1}\left(i\right)$ to final states $a_{\text{end}}\left(i\right)$ and $b_{\text{end}}\left(i\right)$. The following equation models the transition:

(25) $$x\left(t,i\right)=x_{\text{end0}}\left(i\right)+\left[x_{1}\left(i\right)-x_{\text{end0}}\left(i\right)\right]\frac{1+e^{-k_{x0}\left(i\right)\left(1-t_{0}\right)}}{1+e^{-k_{x0}\left(i\right)\left(t-t_{0}\right)}}.$$

In this equation, the parameters $k_{a0}\left(i\right)$ and $k_{b0}\left(i\right)$ are calibrated using a least square approximation of the original RICE50+ values in Gazzotti et al. (2021). The values of $x_{1}\left(i\right)$ and $x_{\text{end}}\left(i\right)$ are derived from the original RICE50+ values. The term $t_{0}$ is set to 4.5. Furthermore, to introduce uncertainty, the inputs $k_{a}\left(i\right)$ and $k_{b}\left(i\right)$ are defined as $k_{a0}\left(i\right)+k_{a}$ and $k_{b0}\left(i\right)+k_{b}$, respectively. Here, $k_{a}$ and $k_{b}$ are uncertain inputs assumed to be the same across regions. They model the steepness of the transition to the final values $a_{\text{end}}\left(i\right)$ and $b_{\text{end}}\left(i\right)$. For the sensitivity analysis, the mentioned inputs $a_{\text{end}}\left(i\right)$ and $b_{\text{end}}\left(i\right)$ are considered uncertain. To account for this uncertainty, the stochasticity of these inputs is modeled using multiplicative scaling as $a_{\text{end}}\left(i\right)=a_{\text{end0}}\left(i\right)a_{\text{end}}$ and $b_{\text{end0}}\left(i\right)=b_{\text{end}}\left(i\right)b_{\text{end}}$. By utilizing these equations and parameterizations, we aim to capture the dynamics of the transitions from initial to final states in the sensitivity analysis.

Cumulative emissions affect the climate, changing the temperature anomaly:

(26) $$\Delta T\left(t\right)=\psi \sum_{i\in N}\sum_{\tau =1}^{t}E_{\text{TOT}}\left(\tau ,i\right).$$

In the equation, ψ is the transient climate response to emissions and is one of the most important values in climate research (Intergovernmental Panel On Climate Change, 2023). It is uncertain and is one of the inputs of our model.

The impact function determines how the temperature affects the economy. Several possible impact functions are available in the literature (Nordhaus, 1992; Burke et al., 2015). We model damages using the impact function from (Kalkuhl & Wenz, 2020), described by the equation:

(27) $$\begin{array}{ccc} \delta (t,i) & = & \kappa_{1}\left(T\left(t,i\right)-T\left(t-1,i\right)\right)\\ & & +\;\kappa_{2}\left(T\left(t,i\right)-T\left(t-1,i\right)\right)T\left(t-1,i\right). \end{array}$$

The variables T(t,i) are the regional temperatures. Both $\kappa_{1}$ and $\kappa_{2}$ are uncertain and are treated as inputs in our sensitivity analysis. The assumption behind the damage function is that climate damages affect economic production, changing consumption.

### 5.2 Inputs and distribution selection

We divide the inputs into two groups: scalar and functional, where functional inputs are time‐ and space‐dependent quantities. For scalar inputs, we assign distributions used in previous studies when available. To illustrate, we assign the distribution assessed in the latest IPCC report (Intergovernmental Panel On Climate Change, 2023) to the input ψ, the transient climate response to emissions. When unavailable, we assign distributions based on rational principles, such as Laplace's principle of insufficient reasons (Laplace, 1840; Gilboa & Marinacci, 2016). Regarding functional inputs, RICE50+ requires three time‐ and space‐dependent quantities: population (P) measured in millions of people, GDP per capita (g) in 2005 USD, and carbon intensity of GDP (σ) in $\mathrm{Gt}{\mathrm{CO}}_{2}$ per 2005 USD, which must be provided for each region. We consider the projections described in Rennert, Prest, et al. (2022), obtained from Bayesian hierarchical models and expert elicitations. Müller et al. (2022) and Raftery and ševčíková (2023) present detailed descriptions of the GDP per capita and population models, respectively. To obtain these projections, we utilize the dataset (Rennert, Prest, et al., 2022). For each country, the dataset includes 1000 projections of population measured in thousands of people, 2000 GDP per capita projections in 2011 USD, and 10,000 global emissions from fossil fuels and industry projections in gigatons of carbon. Population and GDP are then aggregated to the regional level, and linear downscaling as described in Gütschow et al. (2021) is applied to scale the emissions to the regional level. The carbon intensities are evaluated as the ratio of regional emissions to regional GDP.

Because the functional inputs are high‐dimensional, we apply a functional principal component analysis (FPCA) (Hall & Hosseini‐Nasab, 2006) to reduce the dimensionality. The goal is to reconstruct the projections using the principal components (PCs) that explain at least 99% of the total variance in the data. The resulting functional inputs can be represented as

(28a) $$\begin{array}{cc} & \hat{P}\left(t,i\right)=E\left[P\right]\left(t,i\right)+\sum_{j=1}^{n_{P,i}}b_{P,j}\left(t,i\right){\hat{P}}_{j}\left(i\right), \end{array}$$

(28b) $$\begin{array}{cc} & \hat{g}\left(t,i\right)=E\left[g\right]\left(t,i\right)+\sum_{j=1}^{n_{g,i}}b_{g,j}\left(t,i\right){\hat{g}}_{j}\left(i\right), \end{array}$$

(28c) $$\begin{array}{cc} & \hat{\sigma }\left(t,i\right)=E\left[\sigma \right]\left(t,i\right)+\sum_{j=1}^{n_{\sigma ,i}}b_{\sigma ,j}\left(t,i\right){\hat{\sigma }}_{j}\left(i\right). \end{array}$$

This approach reduces the number of functional inputs from 870 to 46 while retaining over 99% of the total variability. The resulting distribution is a nonuniform discrete distribution over the set of PCs' coefficients. The nonuniformity derives from the weights assigned by Rennert, Prest, et al. (2022) to each curve in the dataset. In practice, we sample a curve for each functional input and compute the coefficients of the principal components previously obtained through FPCA. These coefficients are then used to reconstruct the high‐dimensional inputs as defined in Equations (28a)–(28c).

Dimensionality reduction allows us to represent the main characteristics of the dataset concisely, making the evaluation of sensitivity indices faster and easier. Let us consider the available number of emissions samples (10,000) to illustrate why this is beneficial. Given our aim to use the given‐data estimator from Equation (18), we build the partition $X_{i}^{h}$ for h∈1,⋯,H. If each input value were simply an integer between 1 and 10,000 representing the sampled curve, the partitions would be built in this numerical space. However, since these values do not encode actual spatial relationships (as the curves are randomly ordered), a partition containing values such as 1,2,3 would represent three entirely different curves. Instead, by using the space of PCs' coefficients as the input, we capture the meaningful features of the curves within the input space. Then, to evaluate the aggregate indices based on these PCs, we condition the specific principal components and generate the corresponding partitions using K‐means clustering (Cam & Neyman, 1967).

Table 3 summarizes the inputs (23 scalar, 46 functional) names, definitions, and distributions.

**TABLE 3:** Names, distributions, and distribution parameters for the inputs. For the last three inputs, i stands for the region for which we are evaluating the principal component and j is the principal component order.

Symbol	Definition	Distribution	Parameters
dk	Depreciation rate on capital	Uniform	[0.01,0.14]
α	Capital elasticity (production function)	Truncated normal	(0.3,0.051,0,1)
ρ	Initial rate of social time preference	Uniform	[0.1,3]
γ	Inequality aversion	Uniform	[0,2]
η	Marginal utility of consumption elasticity	Truncated normal	(1.36,0.077,0,+∞)
d	Decline rate of land emissions	Uniform	[−0.0335,0.1677]
ψ	Transient climate response	Truncated normal	(1.65,0.395,0,+∞)
$f_{ex}$	2100 forcing of non‐${\mathrm{CO}}_{2}$ GHG	Uniform	[0.5,1.5]
$\kappa_{1}$	First coefficient of the damage function	Normal	(0.00986,0.00999)
$\kappa_{2}$	Second coefficient of the damage function	Normal	(−0.001808,0.00086)
$k_{a}$	Logistic transition speed of a	Uniform	[−0.1,0.1]
$k_{b}$	Logistic transition speed of b	Uniform	[−0.1,0.1]
$a_{end}$	Final value of a	Uniform	[0.9,1.1]
$b_{end}$	Final value of b	Uniform	[0.9,1.1]
A	First abatement exponent	Discrete uniform	{1,2,3}
B	Second abatement exponent	Discrete uniform	{4,5,6}
$k_{l}$	Logistic transition speed of ν	Uniform	[0,1]
$T_{pb}$	Time of full‐convergence to backstop curve	Discrete uniform	[18,28]
g	Initial backstop cost decline	truncated normal	(0.025,0.01,0,1)
$T_{\mu }$	Time of transition to M	Discrete uniform	[18,38]
Δμ	Maximum variation of μ in one time step	Uniform	[0.1,0.3]
M	μ maximum reachable upper bound	Uniform	[1,1.4]
$f_{max}$	Maximum cumulative extraction of fossil fuels	Uniform	[5000,20,000]
${\hat{\sigma }}_{j}\left(i\right)$	jth PC of the regional carbon intensity	Discrete	
${\hat{P}}_{j}\left(i\right)$	jth PC of the regional population	Discrete	
${\hat{g}}_{j}\left(i\right)$	jth PC of regional GDP per capita	Discrete	

The next step is dealing with nonprobabilistic inputs (Figure 2) and defining corresponding scenarios. In our model, these inputs are the cooperation (represented by a boolean value, with 0 meaning noncooperation and 1 meaning cooperation) and the climate target (represented by an exogenous constraint on temperature anomaly). Treating these inputs inside a scenario framework isolates their effects from those of other uncertain inputs, helping to avoid conflating the effects of deterministic policy choices with the influence of probabilistic, uncertain parameters. This separation can make sensitivity analysis results clearer and more interpretable, providing policy‐makers with a more distinct understanding of how variability in economic, technological, or other factors impacts outcomes within fixed policy settings.

We evaluate three climate policy scenarios: the non‐cooperation, the cooperation, and the Paris agreement target. The non‐cooperation and cooperation scenarios are cost‐benefit analyses, meaning that we look for the emissions pathways that can balance the costs of climate change and mitigation. The difference between the two scenarios is in how the regions behave. In the non‐cooperation scenario, regions act in self‐interest. In the cooperation scenario, the regions fully cooperate to get the best possible global welfare. The last scenario, the Paris agreement target, is a cost‐effective analysis. The goal is to find the least‐cost emission pathways consistent with the climate target of 2°C by the year 2100 agreed upon in the Paris Agreement.

### 5.3 Uncertainty quantification

For each scenario, we perform an uncertainty quantification. First, we generate a sample of n=50,000 input realizations from the joint input distribution using Latin hypercube sampling (see Section C for greater details on the numerical implementation). We then conduct Monte Carlo simulations and calculate the quantities of interest: the ${\mathrm{CO}}_{2}$ emissions and the temperature anomaly (yellow boxes in Figure 3).

**FIGURE 4:**
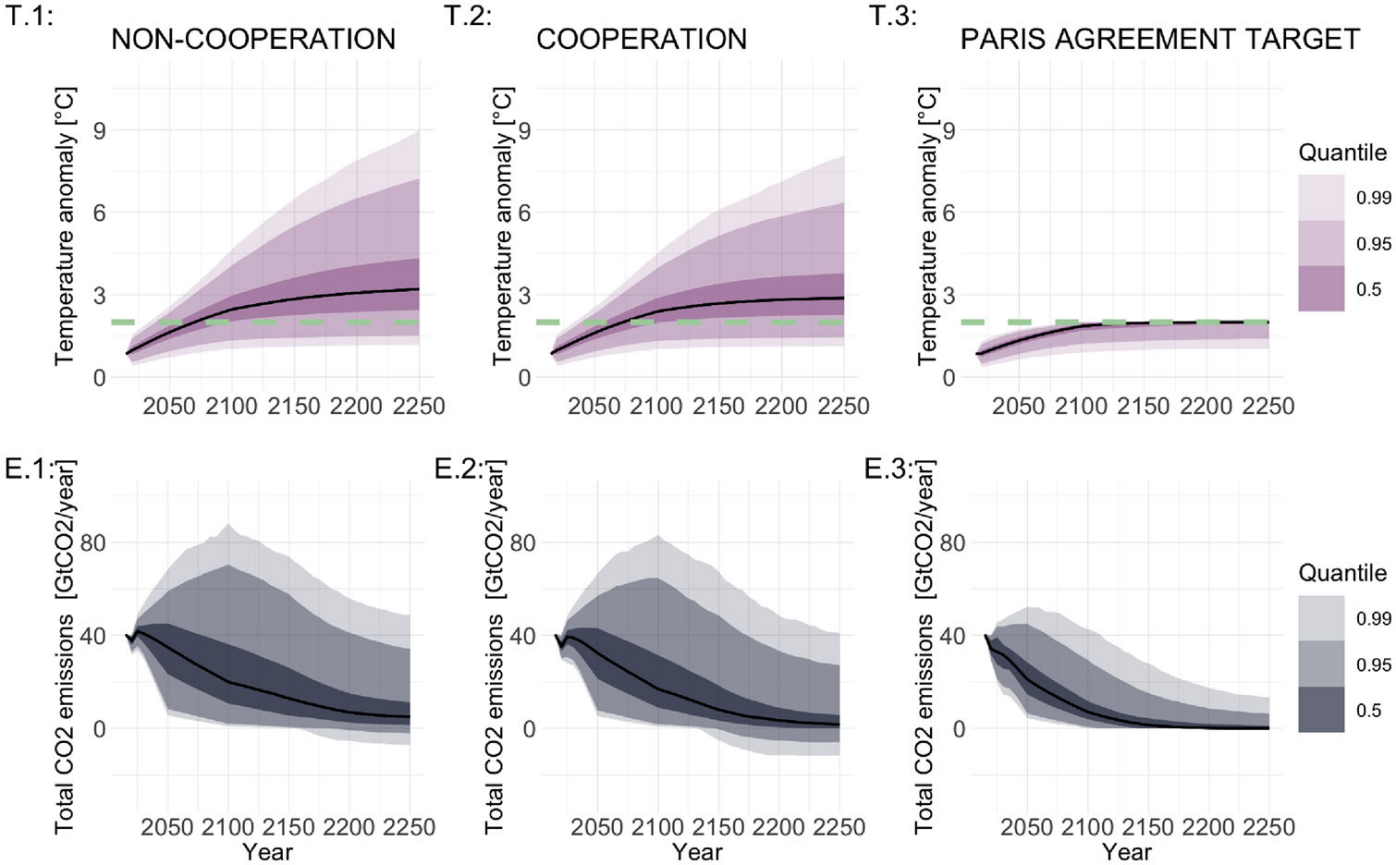
Empirical quantiles for temperature (upper panels) and global ${\mathrm{CO}}_{2}$ emissions (lower panels) for the three climate policy scenarios. The black continuous lines represent the median. The green dotted lines in panels T.1–T.3 are the 2°C threshold.

In the Paris agreement target scenario, 24,420 simulations have a feasible solution. The number increases to 39,250 in the cooperation scenario and to 49,910 in the non‐cooperation scenario. Removing unfeasible realizations induces probabilistic dependencies among the inputs, making the independence assumption no longer valid. This does not pose an issue for the OT‐based indices, as they are well‐defined for dependent inputs. However, these dependencies could present challenges for other methods, such as variance‐based, whose interpretation is clearer under the assumption of input independence.Figure 4 displays the median and empirical quantiles obtained from the Monte Carlo sample of the two quantities of interest. The model output distributions in Figure 4 reveal differences between the non‐cooperation, cooperation, and Paris agreement target scenarios. The temperature medians in the non‐cooperation and cooperation scenarios cross the 2°C threshold around 2070 and show similar behavior until 2100. Thereafter, the difference between the two scenarios becomes more pronounced, with the temperature medians reaching above 3°C in the non‐cooperation scenario (3.21°C in 2250) while remaining below 3°C in the cooperation scenario (2.88°C in 2250). We find differences in the quantile ranges between the cooperation and the non‐cooperation scenarios. Panel T.1 shows that 99% of the non‐cooperation scenario temperatures in the year 2250 lie between 1.2°C and 9°C. In contrast, Panel T.2 shows that the range narrows to [1.15,8] in the cooperation scenario. A similar pattern holds for the 2.5th and 97.5th quantiles. This result indicates that, under parametric uncertainty, international cooperation helps to achieve lower temperature anomaly, but not enough to remain within the limits set in the Paris Agreement. However, only around 10% of realizations in the non‐cooperation scenario remain under the 2°C threshold. This percentage increases to 14% in the cooperation scenario. Thus, if we take uncertainty into account, cooperation alone is not sufficient to achieve the temperature target considered. Even though the model allows for negative emissions before 2100, Panel T.3 highlights that the probability of overshooting in the Paris agreement target scenario is almost zero, meaning that all the obtained trajectories remain under 2°C even before the year 2100. This result depends on the choice of the functional form of the climate damage function: in this study, we perform sensitivity over one specific damage relation (Kalkuhl & Wenz, 2020), but different ones may yield different results, some of which may be consistent with the Paris Agreement target (Gazzotti et al., 2021). This variety highlights that uncertainty is pervasive and is not limited to parametric uncertainty.

Panels E.1–E.3 reveal a similar behavior of the emissions medians across all three scenarios, with a steady decline occurring already before 2030. However, results are highly impacted by uncertainty. In Panels E.1 and E.2, the interquantile range of the emissions in 2100 covers two orders of magnitude. In the non‐cooperation scenario, the 2.5th quantile is around 2 $\mathrm{Gt}{\mathrm{CO}}_{2}$ while the 97.5th quantile is 70.43 $\mathrm{Gt}{\mathrm{CO}}_{2}$. A similar variation range is present in the cooperation scenario, where the 2100 emissions span from 1.74 $\mathrm{Gt}{\mathrm{CO}}_{2}$ (2.5th quantile) to 64.58 $\mathrm{Gt}{\mathrm{CO}}_{2}$ (97.5th quantile). We also observe significant emission variations in the Paris Agreement target scenario, though they naturally decline faster than in the other two cases given that the temperature is in this case constrained to remain below 2°C. This variability prevents us from drawing clear conclusions on the values of output emissions or temperature anomalies under a variety of different climate policy architectures. In this context, understanding and analyzing the sources of output uncertainty becomes crucial.

### 5.4 Global sensitivity analysis

The last step in Figure 3 is sensitivity analysis. First, we investigate the sensitivities of the whole (overall) output, meaning that we consider the entire vectorial response ${\left\{Y_{t,i}\right\}}_{t,i}$, t=1,⋯,58 and i∈{asia,oecd,lam,maf,ref} as our target (Section 5.4.1). We estimate the Wasserstein–Bures indices (Equation 13), calculating their variance‐based (Equation 15) covariance‐based components (Equation 16), as well as the overall importance (Equation 12). We then obtain information on the impact of the input variation on the output mean, variance–covariance matrix, and the whole distribution.

We also carefully consider the process of screening the least relevant inputs. To avoid judging an active input as irrelevant (false negative error), in line with the methodology used by Noacco et al. (2019) and Zadeh et al. (2017), we introduce auxiliary irrelevant inputs. These are auxiliary random inputs independent of the model output. The importance of an auxiliary irrelevant input is theoretically zero by the zero‐independence property. A non‐null value is only due to noise in the finite‐sample estimation process. This value is then a benchmark against which to compare the value of the importance of an input to see whether it is relevant. If the importance index of an input is at or below the threshold, its overall effect will be lower than that of an auxiliary irrelevant variable. Consequently, it can be safely regarded as nonimportant. Note that, within an optimal transport framework, the specific distribution or support of the auxiliary irrelevant variable is not critical, because the OT indices rely on the reordering of the model output based on the input ranking, making the approach distribution‐agnostic.

Next, we analyze the emissions of each region separately (Subsection 5.4.2). Given the complete emission profile ${\left\{Y_{t,i}\right\}}_{t,i}$, we set $i=\bar{ı}$ and we perform the sensitivity analysis of the resulting quantity of interest ${\left\{Y_{t,\bar{ı}}\right\}}_{t}$. For this analysis, we calculate only the OT index $\iota^{W_{2}^{2}}$ because we are interested in how the input importance shifts across the regions. Then, we compute the sensitivity OT indices sensitivity maps: we fix time ($\bar{t}$) and region ($\bar{ı}$), and focus our attention on the scalar quantity $Y_{\bar{t},\bar{ı}}$ (Subsection 5.4.3). This analysis yields a more granular understanding of the importance of the inputs, informing on how, when, and where an input becomes important. Lastly, we compare the results obtained with the multivariate output ${\left\{Y_{t,i}\right\}}_{t,i}$ against two different scalar quantities: the total cumulative emissions and the global emissions in the year 2100 (Subsection 5.4.4). The goal is to show the capability of the multivariate approach to obtain more refined insights about the model.

#### 5.4.1 Global results

**FIGURE 5:**
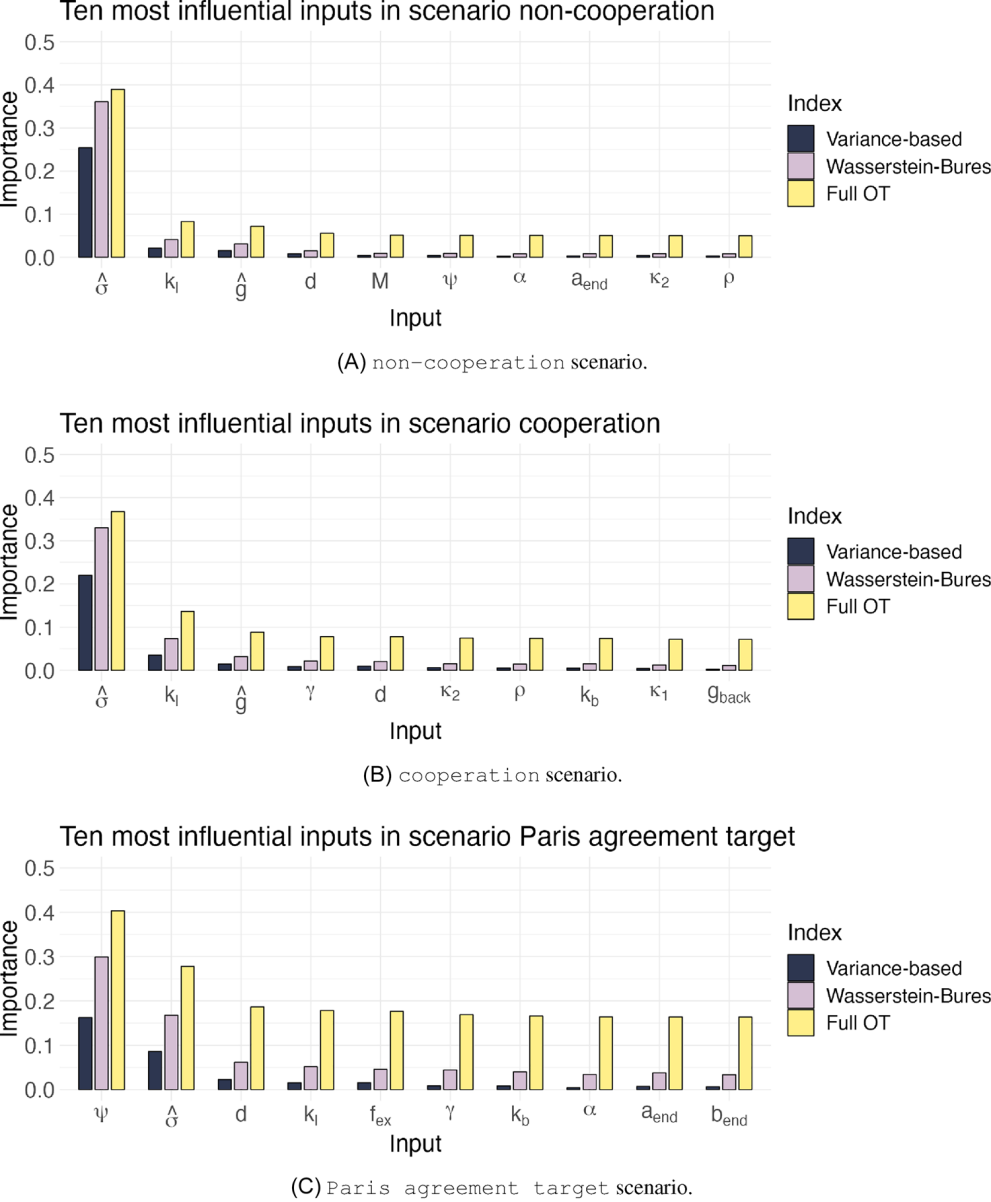
Ten most influential inputs per scenario, ranked according to the OT index ($\iota^{W_{2}^{2}}\left(Y,X\right)$).

As described in Section 4, we first study the sensitivities of the complete emissions profile ${\left\{Y_{t,i}\right\}}_{t,i}$. Figure 5 presents the 10 most important inputs for ${\mathrm{CO}}_{2}$ emissions in the three scenarios. The inputs are ranked according to the OT indices ($\iota^{W_{2}^{2}}$). The three sensitivity indices have a consensus on many of the most important inputs in all scenarios. In the non‐cooperation scenario, the indexes agree on the 10 most significant inputs. In the cooperation case, they concur on nine out of 10 inputs, and in the Paris agreement target scenario, they agree on eight out of ten inputs.

Figure 5A shows that, in the non‐cooperation scenario, carbon intensity ($\hat{\sigma }$) is the most important input. Its OT index ($\iota^{W_{2}^{2}}\left(Y,\hat{\sigma }\right)$) is 0.39, while the Wasserstein–Bures index ($\iota^{WB}\left(Y,\hat{\sigma }\right)$) is 0.36, and the variance‐based ($\iota^{V}\left(Y,\hat{\sigma }\right)$) is 0.25. The small difference between $\iota^{W_{2}^{2}}\left(Y,\hat{\sigma }\right)$ and $\iota^{WB}\left(Y,\hat{\sigma }\right)$ implies that the statistical properties mostly affected by carbon intensity changes are mean and covariance and only 0.03 of the OT index is due to higher order moments.

Among the other significant inputs, the logistic transition speed of ν, denoted as $k_{l}$, remains important across all scenarios, as it drives the change in the abatement cost multiplier (Equation 24). $k_{l}$ is a highly uncertain input without a direct socioeconomic interpretation. Its high OT index value (0.08) means care is needed in handling and calibrating it. A further noteworthy input is the GDP per capita, $\hat{g}$, with an OT index of 0.07. This result reinforces the conclusions drawn by Marangoni et al. (2017) in their multimodel comparison about the importance of economic development. From the fourth input in importance (d) onward there is a long plateau, with OT indices ranging from $\iota^{W_{2}^{2}}\left(Y,d\right)=0.06$ to $\iota^{W_{2}^{2}}\left(Y,T_{pb}\right)=0.04$. The two least important inputs are the abatement cost exponents, A and B, with OT indices at about 0.03. To better interpret the index range, we generate two samples of 49,910 realizations, one from a normal distribution with mean zero and standard deviation one, and one from a uniform distribution in [0,1], independent from the output ${\left\{Y_{t,i}\right\}}_{t,i}$. The OT index of these auxiliary irrelevant variables has a value of approximately 0.03. Thus, any input with a value of $\iota^{W_{2}^{2}}\left(Y,X_{i}\right)$ lower than 0.03 can be considered irrelevant. In our case, several inputs have OT indices close to 0.04, signaling their little influence on the output. From a policy‐making viewpoint, these results tell us that in a world where all the actors are divided and pursue mainly their interests, the primary tool to reduce global ${\mathrm{CO}}_{2}$ emissions is to decrease carbon intensity through technological innovation.

Figure 5B shows that, in the cooperation case, the most important inputs are the same as in the previous scenario. Carbon intensity remains the most influential one with an OT index of 0.37, a Wasserstein–Bures index of 0.33, and a variance‐based index of 0.22. As in the non‐cooperation scenario, most of its importance is due to its impact on the first two moments of the output distribution, as the Wasserstein–Bures index $\iota^{WB}$ represents almost 90% of the OT index $\iota^{W_{2}^{2}}$. The logistic transition speed ($k_{l}$) increases its relevance ($\iota^{W_{2}^{2}}\left(Y,k_{l}\right)=0.14$) compared to the non‐cooperation scenario, and several other inputs are associated with an OT index greater than 0.07. The GDP per capita ($\hat{g}$) has an OT index of 0.09. The inequality aversion (γ) is also important in this case ($\iota^{W_{2}^{2}}\left(Y,\gamma \right)=0.08$), and so is the decline rate of land emissions d ($\iota^{W_{2}^{2}}\left(Y,d\right)=0.08$), the damages (both $\kappa_{1}$ and $\kappa_{2}$ have OT indices higher than 0.07), and the initial rate of social time preference ρ ($\iota^{W_{2}^{2}}\left(Y,\rho \right)=0.07$). Notably, this is a value‐laden and thus controversial parameter which has already been discussed in the literature (Anderson et al., 2014). Its value determines how much we discount the welfare of future generations compared to the current ones. A high discount rate means we care little about the future, encouraging emissions and high near‐term consumption. The choice of this parameter, also related to other economic preferences, has been the subject of intense academic debate, which we cannot summarize here. It suffices to say that the choice of social discount rate matters for the outcomes of climate–economy models not just for the case of benefit‐cost analysis but also for emission pathways obtained through cost‐effective scenarios meeting climate policy targets, and that our distribution, centered around 1.5%, is in line with the most recent estimates.

To set a threshold value for identifying irrelevant variables, we generate two samples of 39,250 realizations, one from a normal distribution with zero mean and unitary variance and one from the uniform distribution in [0,1]. The OT indices of the corresponding auxiliary irrelevant inputs are about 0.03, as in the previous case. Excluding A, B, $T_{\mu }$, and $T_{pb}$, all the remaining inputs have OT indices greater than 0.07. Given the threshold value of 0.03, we can conclude that the output emissions are sensitive to the variation of several inputs in the cooperation scenario.

In the Paris agreement target scenario (Figure 5C), the most important input is the transient climate response to emissions (ψ), a parameter indicating how responsive is temperature to cumulative emissions. Its OT index is about 0.40, its Wasserstein–Bures index is about 0.30, and its variance‐based index is about 0.16. The importance of the climate sensitivity parameter is logical given the need to comply with a given temperature goal. This crucial climate parameter is deeply uncertain. Similarly, the climate contribution of non‐${\mathrm{CO}}_{2}$ greenhouse gases $f_{ex}$ is highly important according to the OT index ($\iota^{W_{2}^{2}}\left(Y,f_{ex}\right)=0.18$). This input is of a similar nature as ψ, and subject to intense negotiations, especially for potent greenhouse gases such as methane. The carbon intensity $\hat{\sigma }$ ($\iota^{W_{2}^{2}}\left(Y,\hat{\sigma }\right)=0.28$), decline rate of land emissions d ($\iota^{W_{2}^{2}}\left(Y,d\right)=0.19$), inequality aversion γ ($\iota^{W_{2}^{2}}\left(Y,\gamma \right)=0.17$), and logistic transition speed $k_{l}$ ($\iota^{W_{2}^{2}}\left(Y,k_{l}\right)=0.18$) remain highly significant, displaying a similarity between this scenario and the previous one.

We then conduct the same test as in the non‐cooperation and cooperation scenario. We create two sets of 24,420 observations, one drawn from a normal distribution with zero mean and unitary variance and another from a uniform distribution between 0 and 1. The OT index for the two exogenous inputs is 0.08. Thus, inputs A and B, the exponents of the abatement costs, are almost irrelevant, as we record $\iota^{W_{2}^{2}}\left(Y,A\right)=\iota^{W_{2}^{2}}\left(Y,B\right)=0.08$. Inputs $T_{\mu }$ and $T_{pb}$ are slightly more important, with OT indices at about 0.12 and 0.13, respectively, while all the other inputs have OT indices greater than 0.16.

Similarly to the cooperation and non‐cooperation scenarios, the Wasserstein–Bures and variance‐based indices show a ranking similar to the OT indices. However, they underestimate the input importance even more than in the previous scenarios. For instance, the variance‐based indices $\iota^{V}\left(Y,X_{i}\right)$ of the ten most important inputs are, on average, only 12% of the corresponding $\iota^{W_{2}^{2}}\left(Y,X_{i}\right)$. For comparison, the corresponding values in the other two scenarios are 17% and 16%. This means that the contribution to output variation is often not captured by the sole impact on the output variance.

In all scenarios, the rankings reveal that output emissions depend on a heterogeneous set of inputs. Important inputs belong to diverse modeling areas: socioeconomic projections ($\hat{\sigma }$, $\hat{g}$), technological projections ($k_{l}$), physical system parameters (ψ), political parameters (γ), and variables that lie at the interface of the different sides ($\kappa_{1}$, $\kappa_{2}$). Thus, when modeling future emissions, it is not sufficient to build narratives on socioeconomic projections (like the shared socioeconomic pathways (SSPs), Riahi et al., 2017) or emissions (like the representative concentration pathways (RCPs), van Vuuren et al., 2011). An effort is needed to thoroughly account for all the relevant uncertainties.

#### 5.4.2 Regional results

**FIGURE 6:**
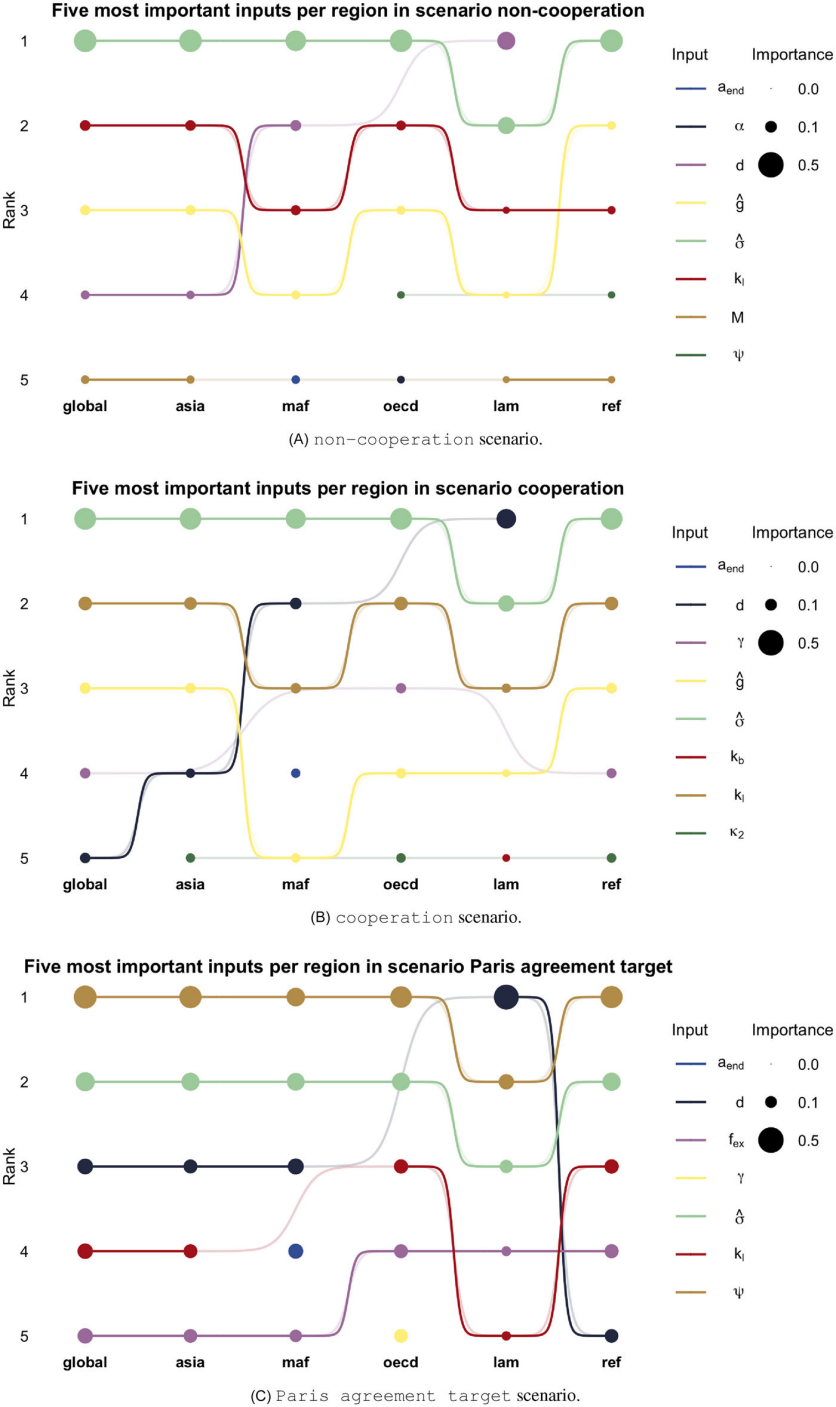
Five most influential inputs per region in the three scenarios according to the OT indices. The dot size is proportional to the importance of the input. The label “global” refers to the rankings described in Subsection 5.4.1

One of the most distinctive features of the RICE50+ model is its high level of regionalization. We examine the results of GSA at the regional level. We focus our analysis on the overall OT indices for the sake of space since the rankings are consistent also with the Wasserstein–Bures and variance‐based indices. Figure 6 displays one bump chart per scenario. The plots show the five most important indices for each of the five regions. The quantities of interest for this sensitivity analysis are the regional emissions ${\left\{Y_{t,\bar{i}}\right\}}_{t}$. The label “global” refers to the rankings described in Subsection 5.4.1. The regions are on the horizontal axis of each chart, and the input importance is on the vertical axis. Each input is associated with a color. The point size represents its importance in the specific region. Dots are connected by lines showcasing the differences between regional rankings.

A first visual impression gained by the charts is a significant consistency of the key drivers of uncertainty across regions. In the non‐cooperation scenario (Figure 6A) and the cooperation scenario (Figure 6B), the carbon intensity ($\hat{\sigma }$) ranks first or second across all regions. Its OT index ranges from 0.22 for Latin America to 0.39 for Asia. Similarly, Figure 6C shows that the transient climate response to emissions (ψ) exhibits high importance for all regions in the Paris agreement target scenario, with an OT index ranging from 0.18 (Latin America) to 0.38 (Asia).

However, a closer examination reveals specific sensitivities unique to each region. Latin America (lam) and the Middle East and Africa (maf) exhibit high sensitivity to the decline rate of land emissions d in all scenarios. This is because tropical forests in these regions host significant amounts of carbon. For the Middle East and Africa, $\iota^{W_{2}^{2}}\left(Y,d\right)$ is 0.09 in the non‐cooperation scenario, 0.10 in the cooperation one, and 0.19 in the Paris agreement target. For Latin America, $\iota^{W_{2}^{2}}\left(Y,d\right)$ is 0.25 in the non‐cooperation scenario, 0.29 in the cooperation one, and 0.48 in the Paris agreement target.

The inequality aversion (γ) affects the emissions projections of the OECD region, which has the highest GDP per capita. The $\iota^{W_{2}^{2}}\left(Y,\gamma \right)$ indices of 0.08 in the cooperation scenario, and 0.14 in the Paris agreement target one indicate a relevant impact on the OECD emissions. Additionally, the emissions projections of the wealthiest regions (OECD, Asia, and Reforming Economies) appear particularly sensitive to the damages (represented by $\kappa_{2}$), which ranks fifth in all the respective rankings in Figure 6B.

**FIGURE 7:**
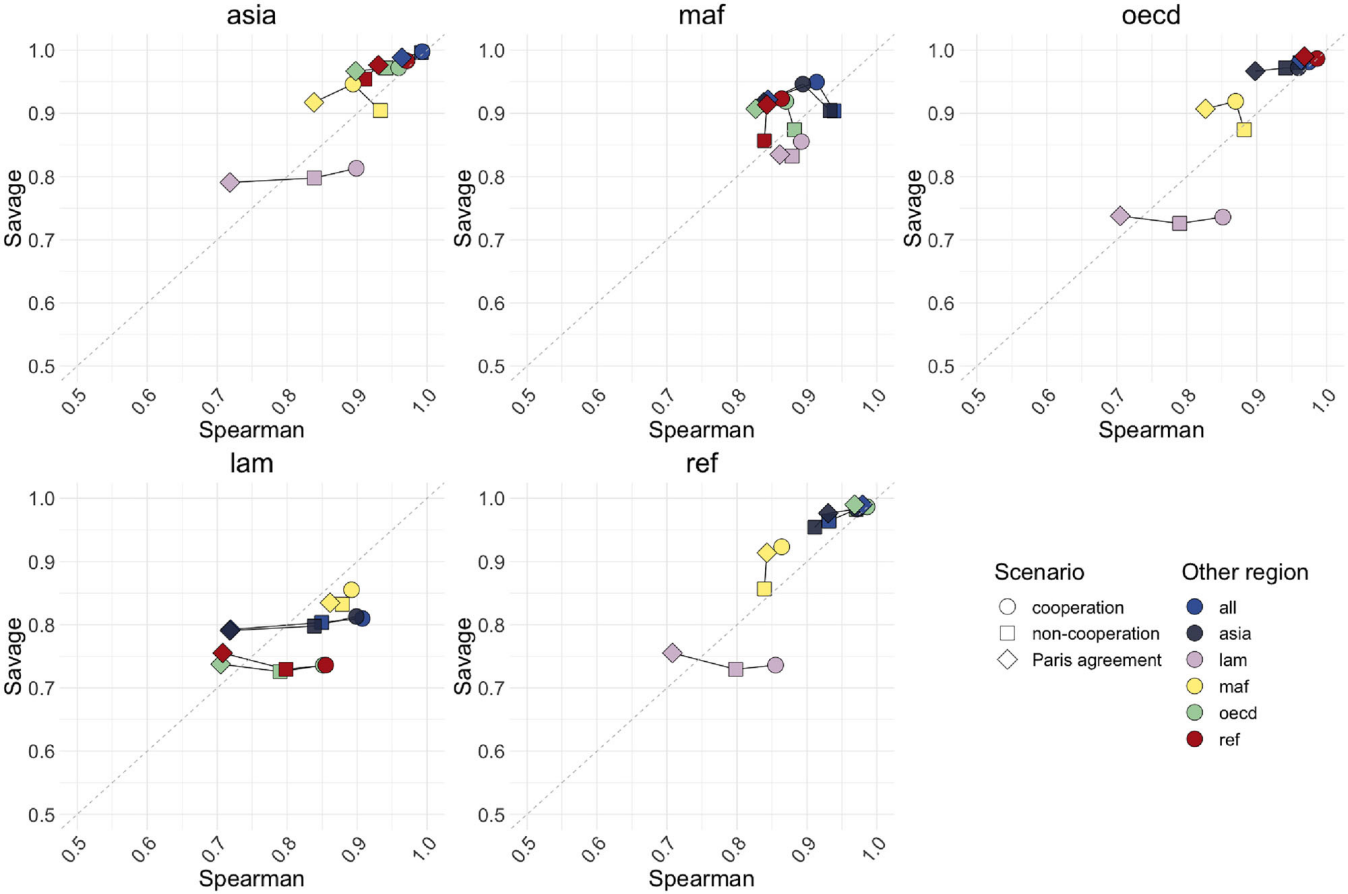
Spearman and Savage correlation ratios evaluated for each pair of regional rankings. The black lines link together the indices evaluated for the same pair of rankings across the different scenarios.

We employ the Spearman and Savage score correlation coefficients (Iman & Conover, 1987) to provide a quantitative and interpretable ranking comparison. The simultaneous use of these two indices allows us to understand whether a ranking agreement is at the level of the most important inputs. Let $\rho_{Sp}\left(r_{1},r_{2}\right)$ and $\rho_{Sa}\left(r_{1},r_{2}\right)$ be the Spearman and Savage correlations between two rankings $r_{1}$ and $r_{2}$, respectively. If $\rho_{Sp}\left(r_{1},r_{2}\right)<\rho_{Sa}\left(r_{1},r_{2}\right)$, the agreement among the top‐ranked inputs is higher than the average agreement. Conversely, if $\rho_{Sp}\left(r_{1},r_{2}\right)>\rho_{Sa}\left(r_{1},r_{2}\right)$, the agreement mainly concerns the less important inputs. Figure 7 shows the results of this analysis. The *x*‐axis represents the Spearman correlation ($\rho_{Sp}$), while the *y*‐axis represents the Savage scores for different regions. Each dot on the chart corresponds to a pair $\left(\rho_{Sp},\rho_{Sa}\right)$. The color represents the region's coupling, and the shape corresponds to the scenario under which the ranking is computed.

The chart shows an overall ranking agreement, with all Spearman correlations and Savage scores above 0.7. However, a general downward trend in Spearman correlation is evident when fixing the climate target (diamond‐shaped dots), especially for the Latin America (lam) region. The average Spearman correlation between Latin America and other regions' rankings (including the “global” ranking) is 0.83 in the non‐cooperation scenario, 0.88 in the cooperation one, and it drops to 0.77 in the Paris agreement target one. However, this trend does not hold for the Savage score correlations. The mean Savage score for Latin America oscillates between 0.78 (non‐cooperation) to 0.80 (Paris agreement target). Indeed, the climate target moves the points from the half‐plane $\rho_{Sp}\left(r_{1},r_{2}\right)>\rho_{Sa}\left(r_{1},r_{2}\right)$ to $\rho_{Sp}\left(r_{1},r_{2}\right)<\rho_{Sa}\left(r_{1},r_{2}\right)$. This shift indicates that, in the Paris agreement target scenario, a high degree of agreement remains on the most important inputs. Nevertheless, the average regional ranking tends to differ more, signaling that each region has specific needs and behaviors.

A cluster of three regions (Asia, OECD, and Reforming Economies) exhibits cohesive rankings across all scenarios while the remaining two regions (Latin America (lam) and Africa and Middle East (maf)) display slightly more specific rankings. These results are consistent with the rankings in Figure 6.

Combining the results from Figures 6 and 7, we gain valuable insights into the consistency and regional variations in the influential inputs hierarchy, enhancing our understanding of the drivers of ${\mathrm{CO}}_{2}$ emissions trajectories in different scenarios and regions.

#### 5.4.3 Sensitivity maps

**FIGURE 8:**
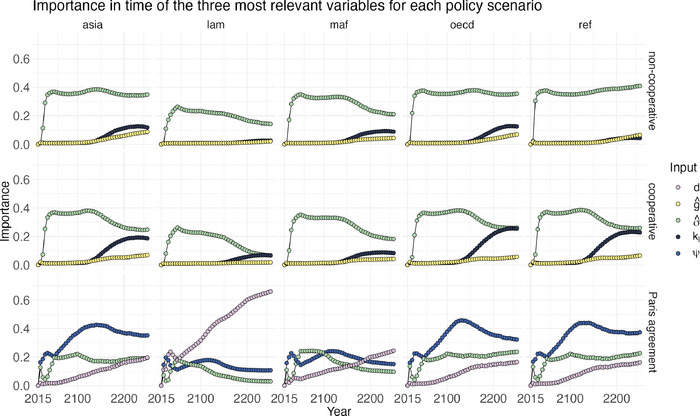
Sensitivity maps of the first three inputs per importance in the global ranking in Subsection 5.4.1.

As pointed out in Section 4, accompanying the multivariate output analysis with a univariate analysis produces additional insights. We discuss how to merge the insights from a multivariate GSA on carbon pathways with the information given by time and regional sensitivity maps. We illustrate the importance over time and region of the three most important inputs for each policy scenario, described in Subsection 5.4.1. Figure 8 represents on the *x*‐axis the year and on the *y*‐axis the importance of the three inputs evaluated on yearly emission for each region and scenario combinations. The time period spans from 2015 to 2250. In other words, each dot is the OT index of an input $X_{j}$ and the emissions of the region i at time t, $\iota^{W_{2}^{2}}\left(Y_{t,i},X_{j}\right)$.

Concerning the non‐cooperation scenario, Figure 8 shows a similar importance behavior in all regions of the three inputs considered. The main difference is in the change in time of the carbon intensity $\hat{\sigma }$ OT index. $\iota^{W_{2}^{2}}\left(Y_{t,i},\hat{\sigma }\right)$ remains relatively stable even beyond 2100 for Asia, OECD, and Reforming Economies. For Latin America, the OT index decreases from $\iota^{W_{2}^{2}}\left(Y_{2050,lam},\hat{\sigma }\right)=0.26$ to $\iota^{W_{2}^{2}}\left(Y_{2250,lam},\hat{\sigma }\right)=0.14$. Similarly, for Middle East and Africa, it reduces from $\iota^{W_{2}^{2}}\left(Y_{2050,maf},\hat{\sigma }\right)=0.35$ to $\iota^{W_{2}^{2}}\left(Y_{2250,maf},\hat{\sigma }\right)=0.21$.

The other two important inputs for the complete emissions profile, $k_{l}$ and $\hat{g}$, are almost nonimportant in the near future but become increasingly important over time. We focus on emissions in Asia to better understand this concept. The logistic transition speed of ν, $k_{l}$, has an OT index of 0.12 in the regional analysis described in Subsection 5.4.2. However, its importance for emissions before the year 2130 is around 0.01. Then, it increases up to a peak of 0.13 in 2250. $\iota (Y_{t,asia},\hat{g})$ has a similar behavior. The OT index for the regional emissions is 0.08, but this importance is not evenly distributed in time: we have $\iota^{W_{2}^{2}}\left(Y_{t,asia},\hat{g}\right)=0.01$ for t between 2015 and 2125, growing thereafter up to 0.09 in 2250.

The second row represents the indices in the cooperation scenario. Near‐future importance is almost the same as in the non‐cooperation scenario, and the long‐term trends are similar but more pronounced. The picture changes when looking at the Paris agreement target scenario. As we can see, the most important input for the complete output, ψ, has a smaller peak in importance between 2025 and 2035 and a second peak between 2100 and 2150. The carbon intensity ($\hat{\sigma }$) is more important between 2050 and 2100 when the temperature constraint becomes effective. Then, its importance decreases or stays the same further in time. The decline rate of land emissions, d, exhibits a different behavior. Its importance increases in the long term, becoming the most important input for two regions after 2200.

**FIGURE 9:**
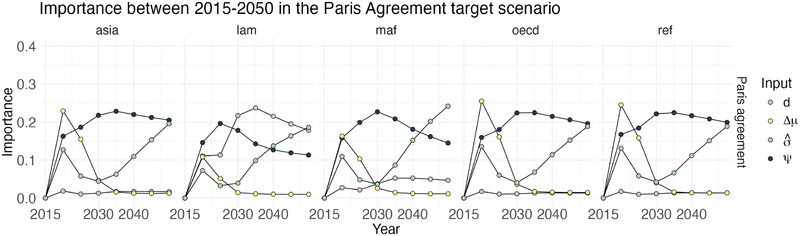
Sensitivity maps between 2015 and 2050 in the Paris Agreement target scenario.

In the Paris Agreement target scenario, the sensitivity indices exhibit noticeable variability from 2020 to 2050, followed by a smooth trajectory. Figure 9 highlights the inputs that reach an importance of at least 0.05 during this period. We identify a long‐term dynamic starting from 2055 and a short‐term one from 2015 to 2050. The long‐term dynamics show a static (asymptotic) behavior and can be analyzed as a whole. The short‐term dynamics display great variability in the importance indices, suggesting the need for a more granular examination of the individual time steps. This difference is driven by two elements in the model structure: first, as in DICE, the upper bound of the mitigation rate (μ) begins a linear shift from 1 to the stochastic value M; second, time is needed for abatement costs to transition toward their long‐run backstop values. These structural shifts explain much of the observed differences. Certain constraints in the model, particularly during the initial time steps, play a significant role as well. For example, the maximum allowable variation of μ (Δμ) is the most influential input in 2020 for four out of five regions, with sensitivity indices $\iota^{W_{2}^{2}}\left(Y_{2020,i},\Delta \mu \right)$ ranging from 0.11 to 0.26. However, its importance decreases steadily after this initial period, reflecting the evolving dynamics of the system as it progresses beyond 2050.

Overall, these findings offer complementary insights to the ones of the previous section, indicating how input importance varies over time and across regions. We can use them to identify relevant time horizons for planning interventions to mitigate the effects of climate change without, with the results of the multivariate analysis providing a reference background.

#### 5.4.4 Comparing univariate and multivariate quantities of interest

A significant improvement in our research lies in the possibility of studying a multivariate quantity of interest. This advantage is particularly relevant when dealing with an inherently multivariate output. However, a multivariate quantity can be summarized in a scalar target differently, forcing the analyst to select only a part of the available information. To illustrate, we compare the results of Section 5.4.1 with the results obtained by synthesizing total emissions in two summary quantities.

**FIGURE 10:**
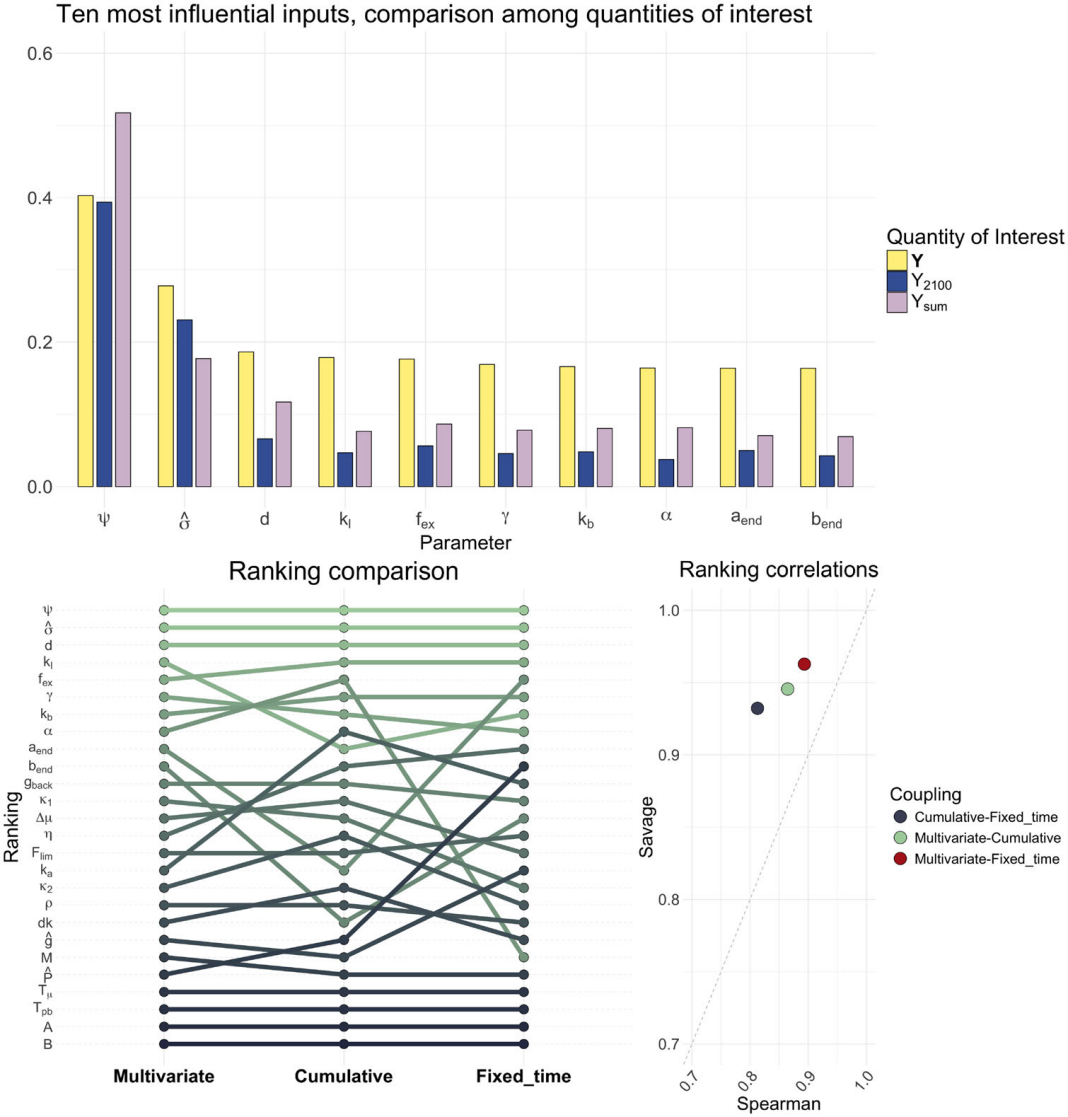
Comparison between the rankings obtained by looking at the multivariate emissions and the two scalar quantities of interest. The label “Multivariate” refers to Y, “Cumulative” to $Y_{sum}$, and “Fixed_time” to $Y_{2100}$.

The first is global cumulative emissions $Y_{sum}=\sum_{t=1}^{T}\sum_{n\in N}Y_{t,n}$. Cumulative emissions are linearly related to temperature increases, and they have already been used in the literature (Marangoni et al., 2017; Bosetti et al., 2015). The second quantity of interest is the global emissions in 2100, $Y_{2100}=\sum_{i\in N}Y_{2100,i}$. Fixed‐year emissions are another value of interest, and they have already been studied in the literature (Anderson et al., 2014). Figure 10 compares the rankings obtained for the multivariate and scalar quantities of interest. In the top panel, similar to those presented in Subsection 5.4.1, we show the 10 most influential inputs for the multivariate and the two scalar outputs as ranked by $\iota^{W_{2}^{2}}\left(Y,X_{j}\right)$. While we record an overall agreement, some differences are worth underlining. For output $Y_{sum}$, the transient climate response to emissions (ψ) is much more important than the other inputs, whereas for Y and $Y_{2100}$ this gap in importance is less pronounced. To quantify, $\iota^{W_{2}^{2}}\left(Y_{sum},\psi \right)=0.52$, $\iota^{W_{2}^{2}}\left(Y_{2100},\psi \right)=0.39$, and $\iota^{W_{2}^{2}}\left(Y,\psi \right)=0.40$ while the second most important input, $\hat{\sigma }$, has OT indices $\iota^{W_{2}^{2}}\left(Y_{sum},\hat{\sigma }\right)=0.18$, $\iota^{W_{2}^{2}}\left(Y_{2100},\hat{\sigma }\right)=0.23$, and $\iota^{W_{2}^{2}}\left(Y,\hat{\sigma }\right)=0.27$. Thus, considering this quantity of interest, we would have overestimated the importance of the climate response to emissions at the expense of other important inputs.

The parallel coordinate plot in Figure 10 shows additional discrepancies. The inputs are ranked from the most important (at the bottom) to the least important (at the top), and lines connect the corresponding positions of each input in the three rankings. Two inputs ($a_{end}$ and $b_{end}$), which are ranked in the top 10 in the multivariate case, are ranked significantly lower for $Y_{sum}$ (16th and 19th, respectively) and $b_{end}$ is in a low position also for $Y_{2100}$. We also note ranking differences between the two scalar quantities of interest. In particular, α is highly important for $Y_{sum}$ (5th) but of little importance for $Y_{2100}$ (21st). On the other hand, the population ($\hat{P}$) is in the 10th position for $Y_{2100}$ but is significantly less important, 20th, for $Y_{sum}$.

We quantify these discrepancies using the Spearman and Savage correlation ratios introduced in Subsection 5.4.2. The second plot of the second row in Figure 10 represents graphically Spearman and Savage correlation indices. The correlations are high for all the ranking pairs. The Spearman and Savage scores correlation coefficients are, respectively, above 0.8 and 0.9. Thus, the agreement is higher for top‐ranked inputs. In fact, the parallel plot shows that the three most important inputs are the same. In summary, the scalar method proves valuable for conducting a quick and greedy analysis of the model, but it falls short of providing a comprehensive and nuanced perspective on the key drivers of uncertainty.

## 6 CONCLUSIONS

Sensitivity analysis is essential to increase transparency and interpretability in risk assessment studies supported by complex numerical models. However, the analysis becomes challenging when the output is multivariate and the setup requires blending uncertainty quantification and scenario analysis. This is the case of the models used, for instance, to inform policy‐making for institutions such as the Intergovernmental Panel on Climate Change.

We presented a holistic approach that accounts for the multivariate nature of model outputs, allows combining uncertainty quantification with scenario analysis, and keeps the computational burden under control. We described the workflow and applied it to quantify the uncertainty and sensitivity in ${\mathrm{CO}}_{2}$ emission pathways, specifically relying on the RICE50+ model.

Overall, the method effectively allows the analyst to blend insights on the regional and time scale with results at the multivariate output level, obtaining complementary insights. Furthermore, OT indices tie together moment‐independent intuition with moment‐dependence (variance‐based) importance measures, providing complete information on the inputs' importance.

When ${\mathrm{CO}}_{2}$ emissions are computed using RICE50+, uncertainty quantification shows that climate mitigation compatible with the Paris Agreement is possible in the two cost‐benefit analysis scenarios but with different probabilities: 10% of the runs in the non‐cooperation scenario remains below the threshold, while the number is higher in the cooperation case, 14%. GSA shows that, at the multivariate level, variability in the ${\mathrm{CO}}_{2}$ emission trajectories is driven by carbon intensity and uncertainty in the physical and political framework—climate sensitivity and inequality aversion. The analysis underlines the importance of the initial rate of social time preference, a controversial input widely discussed in the literature. At the regional level, the sensitivity map reveals zone‐specific nuances, such as the relevant role of land consumption in Latin America. These results imply that, when drawing conclusions from the model outputs, it is fundamental to consider not only the narratives of the future but also the uncertainty within the models. By assessing the sensitivity of a climate–economy model to its inputs, this methodology contributes to making climate decision‐making better informed and building trust in mathematical modeling for high‐stakes problems.

Multiple research directions can follow the present work. On the one hand, the method can be applied to other models within or outside climate risk assessment. Remaining within climate change mitigation, the method can be used to determine the more relevant variables for different models used to compute different quantities of interest, or even to compare the behavior of alternative models in computing the same quantity of interest. Remaining within the same model one can study alternative setups and modeling choices. For instance, using RICE50+, the analyst might be interested in investigating the impact of the regional choice, adopting a higher granularity. Although the sample size has not been an issue in our study, a further research avenue is the generalization of the method in the context of a simulator–emulator approach and in a high‐fidelity/low‐fidelity modeling setup, to allow exploring complex scenarios while keeping computational cost under control.

## CONFLICT OF INTEREST STATEMENT

The authors declare no conflict of interest.

## Figures and Tables

**FIGURE A.1:**
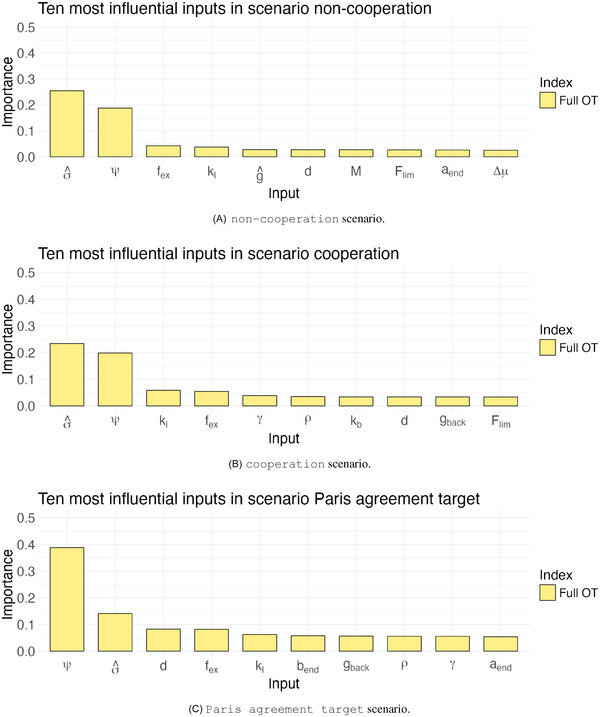
Ten most influential inputs per scenario according to the full OT‐based index for the temperature anomaly.

**FIGURE B.1:**
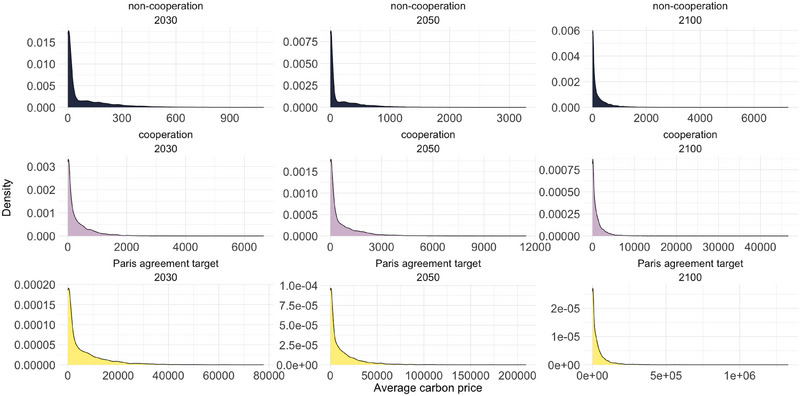
Distribution of the average carbon price in three representative years.

**FIGURE B.2:**
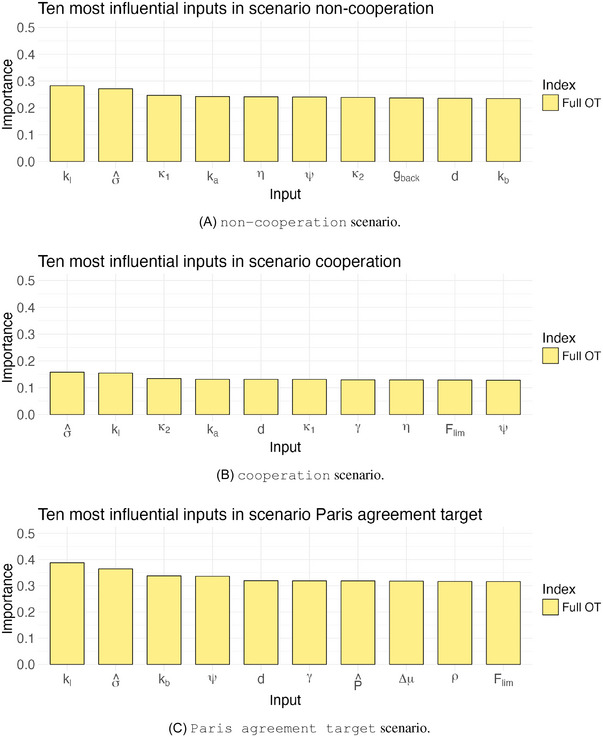
Ten most influential inputs per scenario for the carbon price according to the OT index $\iota^{W_{2}^{2}}\left(Y,X\right)$.

## APPENDIX A SENSITIVITY ANALYSIS OF TEMPERATURE ANOMALY

We report the results for the case in which temperature anomaly (defined by Equation (26)) is the quantity of interest. These results complement the uncertainty quantification discussed in Subsection 5.3. Figure A.1 highlights the 10 most influential inputs according to $\iota^{W_{2}^{2}}$ for each scenario. The carbon intensity $\hat{\sigma }$ and the transient climate response to emissions, ψ, are the most important inputs in all scenarios, non‐cooperation, and Paris Agreement target.

While is also one of the key drivers of uncertainty for the carbon emission profile, the eminent role of ψ for temperature anomaly is expected. In fact, temperature anomaly is expressed as the product of this input times a function of all the others (Equation 26).

## APPENDIX B SENSITIVITY ANALYSIS OF CARBON PRICE

We performed the same sensitivity analysis using the carbon price as our quantity of interest. It is defined in the model as the derivative of the abatement costs (Equation 24).

Figure B.1 represents the distributions of the average carbon price across regions in three sample years, in the three scenarios. It is evident from the figure that the distributions are highly skewed, making the estimation of the sensitivity indices very challenging. Nonetheless, we computed the OT indices to compare the results using this quantity of interest with the results presented in the article.

Figure B.2 reports the 10 most important inputs per scenario according to the OT index ($\iota^{W_{2}^{2}}$). Although the results are noisy due to the skewed distributions, we observe that the most important inputs largely reinforce the conclusions from the article. In the non‐cooperation and cooperation scenarios, the most important inputs are the logistic transition speed of ν and the carbon intensity $\hat{\sigma }$. In the Paris Agreement target scenario, the most important inputs are again $k_{l}$ and $\hat{\sigma }$, with also the logistic transition speed of b ($k_{b}$) and the transient climate response to emissions ψ playing a role. Despite the noisiness of the results, we can still confirm the overall findings presented in the article.

## APPENDIX C CODE

The RICE50+ model is implemented in GAMS, a high‐level modeling system for mathematical optimization. The source code is publicly available in the GitHub repository: https://github.com/witch‐team/RICE50xmodel. The scripts to run Monte Carlo simulations and perform data preprocessing are available in the R programming language upon request to the authors. The simulations were executed on the Zeus high‐performance computer operated by the CMCC foundation, built on Lenovo SD530 nodes interconnected through an Infiniband EDR network. The run‐time changes according to the scenario: 3 h and 55 min for the non‐cooperation scenario, 36 h and 37 min for the cooperation, and 61 h and 27 min for the Paris Agreement target. The model evaluations were performed in parallel processes on 36 cores. The sensitivity indices are evaluated using Octave.

We use part of the Rennert, Prest, et al. (2022) code for the raw data preprocessing. To perform the FPCA, we utilize the fdapace package (Zhou et al., 2022). The sampling is performed using the package FME (Soetaert & Petzoldt, 2010). Additionally, we parallelize the simulations using the future package (Bengtsson, 2021) to enhance computational efficiency. The resulting code is flexible and capable of running a high number of iterations, depending on the specific objectives of the analysis. Furthermore, the structure of the code in R is adaptable to different versions of RICE50+, allowing for modifications to the number of regions and implemented modules.
